# The transcriptome of *Candida albicans* mitochondria and the evolution of organellar transcription units in yeasts

**DOI:** 10.1186/s12864-015-2078-z

**Published:** 2015-10-21

**Authors:** Adam Kolondra, Karolina Labedzka-Dmoch, Joanna M. Wenda, Katarzyna Drzewicka, Pawel Golik

**Affiliations:** Institute of Genetics and Biotechnology, Faculty of Biology, University of Warsaw, Pawinskiego 5a, 02-106 Warsaw, Poland; Institute of Biochemistry and Biophysics, Polish Academy of Sciences, Pawinskiego 5a, 02-106 Warsaw, Poland

**Keywords:** Mitochondria, *Candida albicans*, transcriptome, Introns, tRNA punctuation, Evolution

## Abstract

**Background:**

Yeasts show remarkable variation in the organization of their mitochondrial genomes, yet there is little experimental data on organellar gene expression outside few model species. *Candida albicans* is interesting as a human pathogen, and as a representative of a clade that is distant from the model yeasts *Saccharomyces cerevisiae* and *Schizosaccharomyces pombe*. Unlike them, it encodes seven Complex I subunits in its mtDNA. No experimental data regarding organellar expression were available prior to this study.

**Methods:**

We used high-throughput RNA sequencing and traditional RNA biology techniques to study the mitochondrial transcriptome of *C. albicans* strains BWP17 and SN148.

**Results:**

The 14 protein-coding genes, two ribosomal RNA genes, and 24 tRNA genes are expressed as eight primary polycistronic transcription units. We also found transcriptional activity in the noncoding regions, and antisense transcripts that could be a part of a regulatory mechanism. The promoter sequence is a variant of the nonanucleotide identified in other yeast mtDNAs, but some of the active promoters show significant departures from the consensus. The primary transcripts are processed by a tRNA punctuation mechanism into the monocistronic and bicistronic mature RNAs. The steady state levels of various mature transcripts exhibit large differences that are a result of posttranscriptional regulation. Transcriptome analysis allowed to precisely annotate the positions of introns in the *RNL* (2), *COB* (2) and *COX1* (4) genes, as well as to refine the annotation of tRNAs and rRNAs. Comparative study of the mitochondrial genome organization in various *Candida* species indicates that they undergo shuffling in blocks usually containing 2–3 genes, and that their arrangement in primary transcripts is not conserved. tRNA genes with their associated promoters, as well as GC-rich sequence elements play an important role in these evolutionary events.

**Conclusions:**

The main evolutionary force shaping the mitochondrial genomes of yeasts is the frequent recombination, constantly breaking apart and joining genes into novel primary transcription units. The mitochondrial transcription units are constantly rearranged in evolution shaping the features of gene expression, such as the presence of secondary promoter sites that are inactive, or act as “booster” promoters, simplified transcriptional regulation and reliance on posttranscriptional mechanisms.

**Electronic supplementary material:**

The online version of this article (doi:10.1186/s12864-015-2078-z) contains supplementary material, which is available to authorized users.

## Background

Recent advances in nucleic acid sequencing technology brought a wealth of knowledge on genomics of many interesting and important species that were not among the model organisms of classical molecular biology. Among eukaryotic organisms, ascomycetous yeasts represent a veritable evolutionary laboratory, spanning hundreds of millions years [1] of highly divergent evolution; and their relatively small and compact genomes provide significant insights for comparative genomics [2]. The mitochondrial genome of yeasts is of considerable interest, both for its importance for the cell metabolism, and for its evolutionary history. Nucleo-mitochondrial interactions were shown to contribute to speciation [3–6] and are an important factor in intraspecific variation [7].

The mitochondrial genetic system is a legacy of the bacterial origins of these organelles [8], its coding and regulatory capacity in modern cells is, however, significantly reduced. In yeasts, mtDNA encodes several key subunits of the oxidative phosphorylation (OXPHOS) system – apocytochrome b from Complex III, subunits 1–3 of the cytochrome oxidase (Complex IV), and subunits 6, 8 and 9 of the ATP synthase (Complex V). In a number of hemiascomycetous yeasts the mitochondrial genome also contains genes encoding for subunits of Complex I (NADH dehydrogenase) [9], they were, however, lost in the most extensively studied Saccharomycetaceae family which includes the model species *Saccharomyces cerevisiae* [2]. Complex I genes are also absent from the mitochondrial genome of *Schizosaccharomyces pombe*, which belongs to a separate and distant ascomycetous lineage (Taphrinomycotina). Additionally, yeast mtDNA typically contains genes for two ribosomal RNAs and a complete set of tRNAs. A single ribosomal protein gene, and a sequence encoding the RNA subunit of mitochondrial RNase P are found in some, but not all yeast mtDNAs [10, 11].

Typical features of the mitochondrial genome expression, shared by all Eukaryotes, include polycistronic primary transcription units, often containing both mRNA and functional RNA sequences, that are posttranscriptionally processed by various mechanisms, including tRNA excision by RNase P and tRNase Z (tRNA punctuation) [12], transcribed from simple promoter sequences by a single RNA polymerase related to bacteriophage enzymes [13]. Additional RNA processing mechanisms, described in yeast include exoribonucleolytic trimming [14–17], and processing at specific sequence signals at the 3’ end of mRNAs [18–22].

In spite of a broadly similar core set of encoded genes, the size and organization of yeast mitochondrial genomes vary significantly, from the compact (19 kb) mtDNA of *Schizosaccharomyces pombe,* to the much larger (up to 75–85 kb, depending on the strain) mitochondrial genome of *Saccharomyces cerevisiae*. Frequent rearrangements change the gene order even between closely related species [23–28], and the presence of optional introns [29] further contributes to the evolutionary variability of yeast mitochondrial genomes. The structural variability of yeast mitochondrial genomes also entails considerable differences in the organization of gene expression, with the exception of a few model species there is, however, little experimental evidence concerning functional aspects of organellar gene expression in the majority of these divergent organisms.

Two phylogenetically distant yeast species, *Saccharomyces cerevisiae* and *Schizosaccharomyces pombe* are currently used as model organisms for mitochondrial genetics and molecular biology [19, 30, 31] and have provided a wealth of data on various aspects of organellar biology and the nucleo-mitochondrial interactions. Recently, next generation sequencing (NGS) provided the first insights into the complete transcriptome of *S. cerevisiae* mitochondria [32]. They are also representative of the two major types of the mitochondrial genome expression organization. In *S. cerevisiae* the 35 genes are expressed as 11 separate polycistronic units [32, 33], separated by long noncoding stretches of DNA in the genome, whereas in *S. pombe* the compact mtDNA is transcribed as two [19] primary transcripts. Two long primary transcripts are also a feature of animal mitochondrial genomes, and are thus believed to represent the ancestral state, at least in Opisthokonts [19].

The study of evolution of mitochondrial gene expression is hindered by the paucity of experimental data in species other than the two well-studied model yeasts. *S. cerevisiae* is the only representative of the Hemiascomycetes where systematic studies of mitochondrial gene expression were performed, and for the vast majority of the remaining species the only information available is inferred *in silico* from genomic sequences [10, 26, 34–38]. Outside the Saccharomycetaceae family, only fragmentary mitochondrial gene expression studies were performed in yeasts such as *Yarrowia lipolytica* [39] and *Magnusiomyces capitatus*, where a curious translational bypassing mechanism was recently identified [40]. In spite of extensive studies on the mitochondrial chromosome structure and evolution [27, 28, 35, 38, 41–45], and on mtDNA replication [45–47], there are virtually no experimental data on mitochondrial gene expression in yeasts of the so-called “CTG clade”. This monophyletic clade, distant from the well-known *Saccharomyces* group, consists of species that translate CUG as serine instead of leucine in their nuclear genetic code [41, 48–50] and contains many pathogenic species, including *Candida albicans.*

*C. albicans* is a common commensal of humans and other vertebrates, but in certain situations, particularly in immunocompromised individuals, it can be a source of opportunistic infections and is the most frequent fungal pathogen of humans [51–55]. In addition to the medical interest, *C. albicans* is emerging as an attractive model organism for molecular biology and comparative genomics [46, 51, 52, 56–63]. The study of mitochondrial biogenesis and nucleo-mitochondrial interactions is one of the areas, where it can provide significant insights beyond those gained from work on *S. cerevisiae*, which exhibits many peculiar specific adaptations in its metabolism, many of which evolved in Saccharomycetaceae as a result of the whole genome duplication [64, 65]. Unlike *S. cerevisiae*, *C. albicans* does not exhibit the Crabtree effect, and thus maintains active mitochondrial respiration in the presence of glucose [56], even though it tolerates anaerobiosis and respiratory-deficient mutants are viable [61, 62, 66–68]. It is a petite-negative species, that does not tolerate loss of mtDNA [61, 69], and its mitochondrial genome contains genes encoding Complex I subunits [9, 70], lost from *S. cerevisiae*, as well as from *S. pombe*. Important insights into the mechanisms of mtDNA replication [46, 47, 61] or mitochondrial protein import [62] were obtained using *C. albicans* as a model organism. Additionally, mitochondrial metabolism has been linked to pathogenicity, as respiratory deficient strains were shown to be unable to form biofilms [68], lack virulence [71] or exhibit decreased pathogenicity [72] and increased sensitivity to oxidative stress induced by photodynamic therapy [73].

In spite of this, expression of mitochondrial genes has not been studied to date in *C. albicans*, and the putative promoters, genes, and introns in the mtDNA of this yeast were only inferred from *in silico* comparisons to other species. In order to provide the necessary background for the study of mitochondrial genes in *C. albicans*, and to gain insights into the evolution of mtDNA expression organization in yeasts, we performed systematic analysis of the *C. albicans* mitochondrial transcriptome using deep sequencing, followed by low-throughput analysis of mitochondrial transcripts using RT-PCR, 5’-RACE and Northern hybridization. Our results provided evidence for the composition of polycistronic transcription units, active promoter sequences, processing by tRNA punctuation, split genes, and possible regulatory function of GC-rich sequence elements.

## Results and discussion

### RNA-seq analysis of *Candida albicans* mitochondrial transcriptome

#### Sample collection, library preparation and sequencing

Mitochondria isolated from two commonly used laboratory strains of *C. albicans* (BWP17 [59] and SN148 [57]) were used to prepare RNA sequencing libraries. We also sequenced libraries from total *C. albicans* RNA in order to account for possible artifacts related to the mitochondrial purification protocol. We did not, however, observe any significant differences in mitochondrial transcripts between the two approaches (see Supplementary Results and Discussion in Additional file 1).

Unlike the nuclear mRNAs, but similarly to bacterial transcripts, the primary products of organellar transcription contain a 5’-pyrophosphate [74, 75]. In order to enrich the RNA sequencing products in reads corresponding to the 5’ ends of primary transcripts, an aliquot of each RNA preparation was therefore treated with Tobacco Alkaline Phosphatase (TAP) prior to library preparation. As expected, more reads mapping to regions immediately downstream of identified transcription start sites were observed in libraries prepared from TAP-treated samples, but otherwise the results were very similar to those obtained without treatment with TAP (Additional file 1: Figure S1).

### Reference sequence and read mapping

Reads were mapped to the complete mtDNA sequence of *C. albicans* strain SC5314 [GenBank:AF285261.1] [70]. The 40.4 kb mitochondrial genome of *C. albicans* contains two inverted repeat regions of 6.8 kb with identical nucleotide sequence [76, 77]. In order to maintain the directionality of transcript reads, we removed the second repeat region (IRb) from the reference sequence. In total, about 24 million reads were mapped to the reference mtDNA sequence in all our experiments. When RNA from purified mitochondria was used, more than 50 % of all reads could be unambiguously aligned to the reference sequence. Reads that mapped to more than one position in the reference without the second repeat (less than 1 % of all aligned reads) were discarded. Similar RNA sequencing performance was reported in the study of the *S. cerevisiae* mitochondrial transcriptome [32], where approximately 9 million reads of ~18 million were mapped to the reference. In the human mitochondrial transcriptome [78] only 14 % of RNA-seq reads from mitochondrial preparations aligned to mtDNA. The statistics of the RNA sequencing and mapping of the *C. albicans* mitochondrial transcriptome are presented in detail in Supplementary Results and Discussion (Additional file 1: Table S1).

The majority (>90 %) of reads that did not map to the mtDNA reference could be unambiguously mapped to the nuclear genome sequence, mostly to the highly expressed rRNA and tRNA genes. The most plausible explanation for this result is copurification of cytosolic ribosomes with the mitochondrial outer membrane, which was also observed in the study of human mitochondrial transcriptome [78]. Import of nuclear-encoded transcripts into mitochondria was shown in multiple systems [79] and cannot be excluded in the case of *C. albicans*, but the results obtained using mitochondrial preparations are not sufficient to draw such conclusions.

The aligned reads did not show any nucleotide differences from the reference, indicating that the BWP17 and SN148 strains are identical to SC5314 with respect to their mtDNA nucleotide sequence. Independently cultured BWP17 and SN148 strains gave virtually identical RNA-seq results in all our experiments (Additional file 1: Figure S1). For subsequent analysis we therefore pooled reads from both strains *in silico*, even though they were always grown and used to prepare sequencing libraries separately to control the reproducibility of the results.

**Fig. 1:**
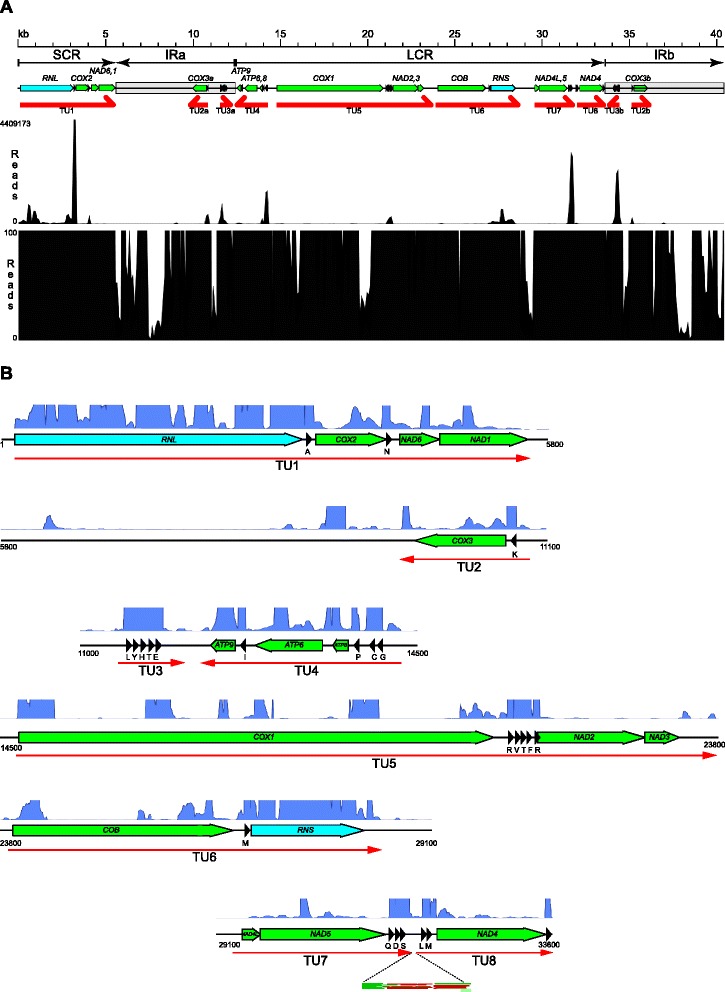
An overview of the mitochondrial genome and transcriptome of *Candida albicans.* **a** Schematic representation of the mitochondrial genome of *C. albicans* with known annotations. The major genomic regions: the short coding region (SCR), the inverted repeats (IRa and IRb), and the long coding region (LCR) are indicated. *C. albicans* mtDNA is mainly found as multiple head-to-tail concatamers, that result in an apparently circular restriction map, even though no actual circles are present [46]. Protein coding genes are shown as *green* arrows, the ribosomal RNA genes (*RNL* and *RNS*) as *light blue *arrows, and tRNA genes as *black* triangles. Labels for tRNA genes were omitted for clarity. *NAD6,1,* etc., means two adjacent ORFS: *NAD6* and *NAD1* expressed from a bicistronic transcript. The major primary transcription units (TUs) are shown as red arrows, indicating direction of transcription. Inverted repeats are marked as shaded boxes. RNA-seq read coverage (based on all ~24 million pooled mapped reads) is displayed in its entirety (upper panel), and clipped at 100 reads to better visualize the expression of mRNA genes (bottom panel). Note that at this scale some short gaps between contiguous transcripts are not visible. **b** RNA-seq read coverage (clipped at 1000 reads) of the regions containing the transcription units. tRNA genes (black triangles) are labeled with one-letter codes of the respective amino acid. The separation of TU7 and TU8 is apparent only when read orientation is considered (inset), see also Fig. 6b

The mitochondrial genome of *C. albicans* is more compact than that of *S. cerevisiae*, and coding regions cover the majority of its sequence. The only larger noncoding stretches (approximately 5.6 kb each) are located in the inverted repeat regions [45, 70, 76, 77]. Consequently, RNA-seq reads map to the majority of the mtDNA sequence, although they do not cover the entire genome (Fig. 1). All the annotated genes are clearly expressed, additional transcriptional activity is also evident in the noncoding regions of the inverted repeats, but it is limited to a few short stretches.

The orientation of strand-specific sequencing reads matches the direction of transcription of a given transcription unit (for transcripts of genes found in the inverted repeat regions this becomes apparent only after the second copy of the repeat is removed from the reference sequence). In total, >96 % of reads map to the appropriate sense strand of the transcript. Reads that map in the antisense orientation constitute only a minor fraction of total reads (~3 %), and they do not form contiguous mappings, suggesting that mainly one strand is transcribed in each of the transcription units, or that any putative antisense (“mirror”) RNAs are very rapidly and efficiently degraded. Whereas many of these rare antisense reads can be artifacts of sequencing or mapping, some of them, clustering near the ends of transcripts or putative processing sites, might indicate the presence of RNA-based regulatory mechanisms (Additional file 1: Figure S2).

### Promoters and transcription units

#### Identification of putative transcription units

**Fig. 2:**
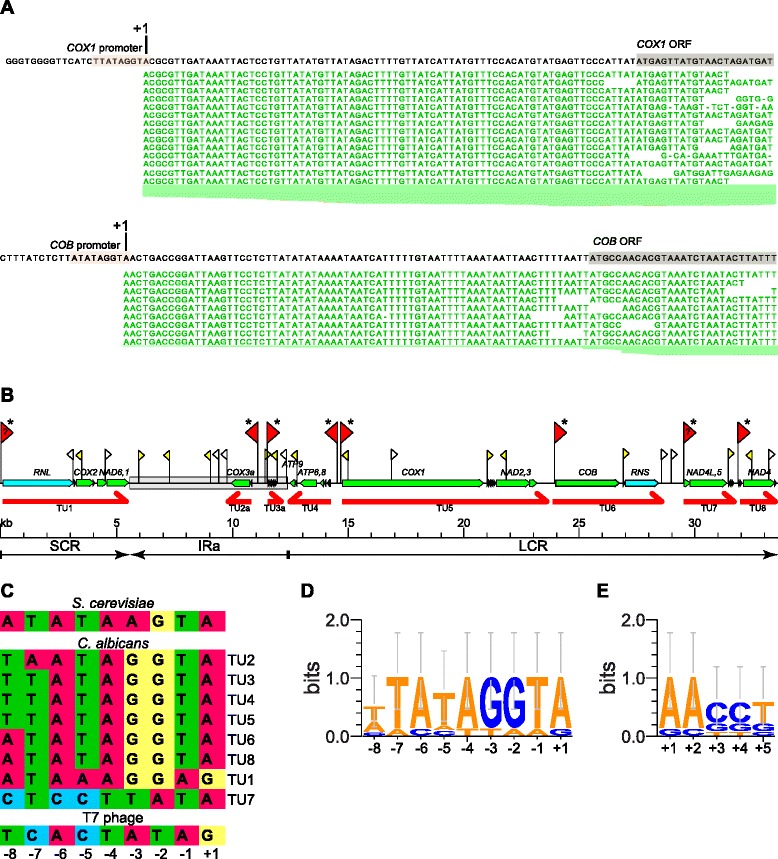
Promoter sites in the *C. albicans* mitochondrial genome. **a** Sequencing reads near the transcription start sites of transcription units TU5 and TU6 (*COX1* and *COB*, respectively). The conserved promoter nonanucleotides, and the start of the open reading frames are boxed. **b** Schematic representation of the mitochondrial genome of *C. albicans* with known genes, major genomic regions and transcription units, annotated as in Fig. 1. The second inverted repeat region (IRb) is identical to IRa in sequence, and was omitted for clarity. Putative promoter sites are marked by flags: larger red flags denote main promoters for transcription units, *yellow* flags correspond to putative additional promoters with some support from RNA-seq reads, and open flags are sequences conforming to the promoter consensus, but with no evidence for activity. Question marks indicate promoters that deviate significantly from the consensus sequence. Asterisks mark transcription start sites independently confirmed by 5’-RACE. **c** Sequences of the promoter sites depicted in (**a**), with the *S. cerevisiae* consensus nonanucleotide mitochondrial promoter and the T7 bacteriophage consensus promoter [88] included for comparison. **d** Sequence logo of the nonanucleotide promoters of the main transcription units (TU1-TU8), and **e** sequence logo of the first 5 nt of the primary transcripts. Logos created using WebLogo 3.4 [143]

Transcription units were identified based on the existence of contiguous overlapping reads from the sense strand, taking into account reads mapping across introns. The 14 protein-coding genes (not including the putative intronic ORFs), two ribosomal RNA genes, and 24 tRNA genes are expressed as eight primary polycistronic transcription units on both strands of the genome (Figs. 1 and 2). The short coding region (SCR) contains one transcription unit (TU1) consisting of *RNL*, *COX2*, flanked by two tRNAs, and the *NAD6*/*NAD1* bicistron. This transcript ends with a GC-rich hairpin sequence at the boundary of the inverted repeat (IRa). The inverted repeats contain two short transcription units, transcribed from opposite strands: TU2 consisting of *COX3* and tRNA-Lys, and TU3 – the only primary transcript containing only tRNAs (five). In addition, two transcripts of unknown function, sharing no similarity with any known mitochondrial gene and devoid of potential open reading frames are observed in the noncoding region of the inverted repeat. Their biological function is currently unknown. The long coding region (LCR) begins with TU4, transcribed in the reverse orientation and containing all three mitochondrial *ATP* genes, as well as four tRNAs. *ATP8* and *ATP6* are translated from a single bicistronic transcript. *ATP9* is separated from the *ATP8,6* bicistron by the tRNA-Ile sequence. The remaining transcription units (TU5-TU8) are expressed in the same orientation, but our results indicate that they have independent promoters, and do not form a single long transcript. The continuity of the identified polycistronic transcription units was confirmed using RT-PCR (Supplementary Results and Discussion, Additional file 1).

Analysis of reads mapping in the 5’ regions of these transcription units allowed us to identify the putative promoter sequences, shown in Fig. 2. The promoter consensus sequence spans nucleotides −8 to +1 (Fig. 2d), and bears general similarity to known nonanucleotide mitochondrial promoters of *S. cerevisiae* [80–82] and other yeasts [83–87]. All but two of the promoters show very tight adherence to the WWATAGGTA consensus (Fig. 2c); the TU1 promoter upstream of the *RNL* gene is the only one, where the +1 nucleotide is G instead of A, whereas the putative transcription start site of TU7, upstream of *NAD4L* significantly deviates from the consensus at several positions, yet has strong experimental support in RNA-seq and 5’-RACE results. Putative promoters of TU7 and TU4 are also the only two where the −5 nucleotide is not T. Interestingly, the unusual TU7 promoter sequence shows some similarity to the bacteriophage T7 promoter [88], with the +1 A of the *C. albicans* transcript corresponding to the −1 position of the T7 consensus (Fig. 2c). Additionally, we observed some conservation of the first 5 nucleotides of the primary transcript (Fig. 2e), with A at positions +1 and +2 in the majority of them and G or C at +3 and +4.

In addition to the promoter sites upstream of the eight identified transcription units, multiple additional sequences conforming to the promoter consensus were found. They are shown in Fig. 2b, and listed in Additional file 2: Table S2. Three such putative promoters, located in the noncoding region of the inverted repeats may be responsible for the transcriptional activity observed in these unannotated regions. Some of the others are located within the transcription units in the sense orientation, upstream of the tRNA gene clusters. As the steady state levels of tRNAs are much higher than those of mRNAs, it is possible that they are auxiliary “booster” promoters ensuring adequate expression of tRNAs located downstream of an mRNA sequence in a polycistronic transcription unit. The sharp increase in the number of reads mapping to tRNAs could, however, also be a result of rapid transcript processing and different stability of tRNA and mRNA sequences. Very high levels of mature tRNAs make detection of unprocessed primary transcripts difficult, and definitive conclusions about any biological relevance of these additional promoter sequences cannot be drawn.

The promoter sequences localized in the transcription unit sequences in an antisense orientation could be involved in regulation, or could simply be inactive. Mapping of RNA-seq reads to the reverse strand of the genomic reference revealed that indeed, some antisense reads do appear downstream of these intragenic reverse-orientation promoters, although the coverage is very low compared to the sense strand expression. For example, a reverse promoter in the *COX1* gene generates antisense reads mapping to the first 336 nt of the transcript (Additional file 1: Figure S2). Regulation by short antisense transcripts was observed in prokaryotic systems [89–91], and it is tempting to speculate that the reverse orientation promoters and antisense reads observed in the *C. albicans* mitochondrial transcriptome hint at the presence of similar mechanisms.

Presence of sequences conforming to the promoter consensus, but not active in transcription was observed in other yeast species, like *S. cerevisiae* [32] and *S. pombe* [19]. High density of potential promoters is a feature of the majority of yeast mtDNAs, although experimental data on their activity exist only in *S. cerevisiae*, *S. pombe,* and as of this study, in *C. albicans*. Assuming that the low density of promoters and the presence of two or three primary transcripts, observed, among others, in *S. pombe* [19], *Candida parapsilosis* [35], filamentous ascomycetes [92–95] and Metazoa [96] is indeed the more ancestral mode of transcription, creation of new transcription start sites had to occur many times in the history of yeast mitochondrial genomes.

#### Identification of the primary transcription start sites by 5’-RACE

We applied the 5’-RACE protocol used to identify transcription start sites in prokaryotic systems [74, 75], including organelles [75], based on the observation that the 5’ ends of primary transcripts, but not RNA processing products, contain a triphosphate group which inhibits adapter ligation, and results in quantitative differences in the obtained PCR product between samples treated with Tobacco Alkaline Phosphatase (TAP) and untreated.

**Fig. 3:**
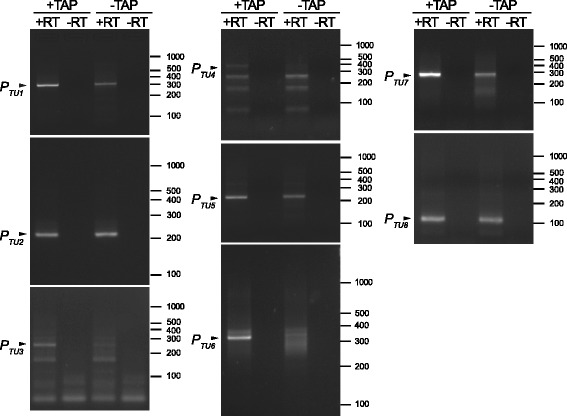
5’-RACE analysis of mitochondrial transcripts. 5’-RACE products amplified from TAP-treated and untreated RNA preparations from purified *C. albicans* mitochondria were separated on 3 % high resolution agarose gels alongside molecular weight markers (O’ Gene Ruler DNA Ladder Mix, Thermo Scientific) and visualized by ethidium bromide staining. Bands corresponding to primary transcript 5’ ends are indicated by arrows and labeled with the name of the respective transcription unit. Positions of bands from the molecular weight markers are indicated on the left side of each gel. The nomenclature of primary transcription units follows Fig. 2b

We applied this strategy to each of the eight primary transcription units (TU1-TU8). Additionally, we attempted to confirm the activity of two putative secondary “booster” promoters localized upstream of tRNA-Arg_1 (tR(UCU)) and *RNS* sequences, inside TU5 and TU6, respectively. Untreated and TAP-treated RNA from purified mitochondria was ligated to an oligonucleotide adapter and reverse-transcribed as described in Methods. 5’-RACE products were visualized on agarose gels (Fig. 3) and also cloned into a plasmid vector after excision from the gel. The inserts (usually 5–20 individual clones) were sequenced to determine the exact 5’ nucleotide, to which the adapter had been ligated.

Analysis of the 5’-RACE products of transcripts containing the *RNL* rRNA sequence indicates that they start at the position identified by RNA sequencing, downstream of the noncanonical promoter, with G as the +1 nucleotide. The amount of amplified product increased significantly in TAP-treated RNA preparation, suggesting that this corresponds to a *bona fide* transcription start site. Similarly, the primary transcription start sites of TU5 (upstream of *COX1*), TU6 (upstream of *COB*) and TU7 (upstream of *NAD4L*) were clearly confirmed by 5’-RACE (Fig. 3a). TU7 is particularly interesting, as its transcription starts at a site that significantly deviates from the nonanucleotide consensus promoter sequence (Fig. 2c). The results of 5’-RACE for this region were, however, unambiguous, with a clear increase in the amplified product after TAP treatment. Additionally, sequencing of the cloned 5’-RACE products resulted in 8/10 clones starting at the predicted +1 position (the remaining two had one nucleotide at the 5’ end truncated) for the TAP treated preparation; whereas only 1 in 5 clones from the untreated preparation started in the predicted region. These results clearly indicate that the unusual TU7 promoter is indeed an active transcription start site.

The results for the transcription units that start with tRNA sequences are more difficult to interpret, as these transcripts are immediately efficiently processed by tRNA excision, and the mature tRNAs are generally stable and present at high steady-state levels. Nevertheless, 5’-RACE amplification of the 5’ terminus of the TU4 transcription unit clearly shows a band corresponding to the predicted molecular weight of the product starting at the position of the indicated consensus promoter, that is apparent only in TAP treated preparations. Three additional bands correspond in size to the predicted products of RNA processing by tRNA punctuation, and they are present in TAP-treated and untreated reactions in comparable amounts (Fig. 3).

In the case of TU2, the major visible amplified product, obtained using starters annealing to the *COX3* coding sequence, corresponds to the processed transcript with the tRNA (tK(UUU)) already excised, as evidenced by the sequence of cloned inserts. The location of the start site of this transcription unit is, however, quite clear, as there is only one putative promoter sequence in the correct orientation in the appropriate upstream region, and the mapping of RNA sequencing reads matches the identified consensus nonanucleotide promoter location. Similarly, in the case of TU8 the sequenced 5’-RACE products correspond to the transcript already processed at the beginning of the second tRNA (tL(UAG)). Both RNA sequencing reads and 5’-RACE analysis indicate, however, that TU8 is transcribed separately from the preceding transcription unit (TU7), and the identified promoter, conforming to the nonanucleotide consensus, is the only plausible candidate for its transcription start site.

The case of TU3, the only transcription unit consisting solely of tRNAs, is more complicated, as there are two potential consensus promoters, one 6 bp, and the other 99 bp upstream of the first tRNA sequence (tL(UAA)). Sequencing of the cloned 5’-RACE products amplified from TAP-treated RNA resulted in two (out of 11) clones starting at the +1 nucleotide of the closer promoter. The remaining clones corresponded to the products of tRNA excision, and no clone with a sequence starting at the upstream promoter was identified. Analysis of RNA sequencing revealed, however, that even though the vast majority of reads start immediately upstream of tL(UAA), there are detectable reads spanning the upstream region and appearing to originate from the first, upstream promoter. It is therefore likely that both promoters upstream of TU3 are active, but it’s the second one that contributes to the bulk of transcription. Interestingly, the steady state level of the second tRNA in this transcription unit (tY(GUA)) is much higher than that of the first one (tL(UAA)). The interplay of the two promoters, together with RNA processing and degradation, could contribute to the observed differential expression of tRNAs encoded in the same transcription unit. Similar multiple promoters have been identified in plant mitochondria [75].

Transcription initiation from the putative promoters located inside the primary transcription units could not be confirmed using RT-PCT (Supplementary Results and Discussion, Additional file 1: Figure S3).

### The mature transcripts, introns, and RNA processing

#### Differences in the expression of mitochondrial genes transcribed as polycistronic primary transcripts

**Fig. 4:**
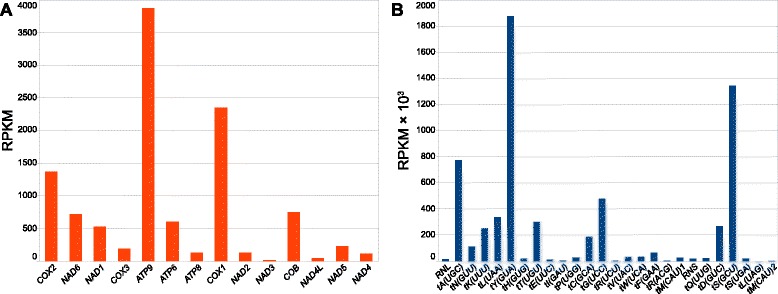
The steady state levels of *C. albicans* mitochondrial RNAs measured in RPKM. **a** Expression of protein coding genes, displayed as RPKM, and **b** transfer RNAs and ribosomal RNAs, displayed as thousands of RPKM. The order of transcripts in each panel corresponds to the order of genes on the genetic map (Figs. 1 and 2b). Reads (obtained from mitochondrial RNA preparations, not treated with TAP), were mapped to the genomic reference sequence with one of the two identical repeat sequences removed to ensure unique counting of reads corresponding to genes localized in those repeats. Data used to generate this figure are available in Additional file 3: Table S3

Counting reads mapping to the annotations in the reference genome allowed to compare the expression levels of different transcripts, calculated as RPKM values. The steady state levels of mitochondrial RNAs vary greatly, sometimes by several orders of magnitude (Fig. 4, Additional file 3: Table S3). Generally, the most abundant transcripts are some of the tRNAs, followed by rRNAs, although five of the tRNAs have lower RPKM values than rRNAs (Fig. 4b). Differences between the RPKM values of different tRNAs can be as large as about two thousand-fold (between tY(GUA) and tL(UAG), Fig. 4b). Differences in expression of protein-coding transcripts are not as large as between noncoding RNAs (Fig. 4a) with *ATP9, COX1* and *COX2* displaying highest RPKM values, and *NAD3* and *NAD4L* the lowest (the difference between *ATP9* and *NAD3* is about 276-fold). The two rRNAs of the small and large subunits of the mitoribosome, encoded by the *RNS* and *RNL* genes, respectively, are expressed at the same level (16 309 and 15 162 RPKM, respectively, about 7 % difference), consistent with the 1:1 stoichiometry of the subunits.

Significant differences between steady state levels of RNAs expressed from a single polycistronic transcription unit were also observed in the *S. cerevisiae* [32] and human [78] mitochondrial transcriptomes, and are most likely due to differences in stability and degradation [32, 78, 97, 98]. Transcriptional attenuation was suggested as an additional mechanism in cases, when the relative transcript abundance decreases in the 5’ to 3’ direction [98], but this does not seem to be the general rule in *C. albicans* mitochondria (Figs. 2b and 4). Additional putative promoter sequences, found inside the primary transcription units described above (Fig. 2b), such as those upstream of tRNA-Ile (tI(GAU)) in TU4, upstream of the cluster of 5 tRNAs downstream of *COX1* in TU5, upstream of the *RNS* gene in TU6 and upstream of the three tRNAs downstream of *NAD5* in TU7, could also play a role in generating differences in expression of genes located on the same main transcription unit by boosting the levels of tRNAs and rRNAs.

Reproducibility of the expression data was confirmed by comparing results obtained from two *C. albicans* strains grown and analyzed separately, as well as mitochondrial and total RNA preparations (Supplementary Results and Discussion, Additional file 1: Figure S1).

#### Mature monocistronic and bicistronic transcripts are generated by tRNA punctuation

The mitochondrial genome of *C. albicans* encodes 14 proteins (not including the putative proteins encoded in introns) that function in the complexes of the OXPHOS system: 7 subunits of Complex I (*NAD1, NAD2, NAD3, NAD4, NAD4L, NAD5, NAD6*), 1 Complex III subunit (*COB*), 3 Complex IV subunits (*COX1, COX2, COX3*), and 3 subunits of the ATP synthase (*ATP6, ATP8*, *ATP9*). Additionally, we confirmed the presence and expression of both ribosomal RNAs of the small and large subunit, encoded by the *RNL* and *RNS* genes, respectively, and the full set of 24 tRNAs (Table 1). One protein coding gene (*COX3*) and six tRNA genes are located in the inverted repeat regions, and thus are annotated twice in the reference genome sequence. Analysis of the tRNA sequencing reads reveals the presence of the non-templated posttranscriptionally added 3’ terminal CCA trinucleotide [99] in all 24 of them, confirming that they are active and processed into mature tRNAs, and allowing precise mapping of the 3’ ends of respective genes. The tRNAs encoded by the *C. albicans* mitochondrial genome are consistent with the genetic code that differs from the standard in using UGA for Trp (instead of stop) [100]. This codon assignment is common in mitochondria of many fungi, protozoa and other organisms, and is described as the “mold, protozoan, and coelenterate mitochondrial code and the Mycoplasma/Spiroplasma code”, also known as “translation table 4”, at NCBI (http://www.ncbi.nlm.nih.gov/Taxonomy/Utils/wprintgc.cgi), but is different from the code used in *S. cerevisiae* and related species [101].

**Table 1:** *C. albicans* mitochondrial tRNAs. Systematic names are according to the guidelines published in the *Candida* Genome Database (http://www.candidagenome.org, [105]). Transcription units are numbered according to Figs. 1 and 2

Common name	Systematic name	Strand	Transcription unit	start position	end position
tRNA-Ala	tA(UGC)mt	+	TU1	3 171	3 242
tRNA-Asn	tN(GUU)mt	+	TU1	4 001	4 072
tRNA-Lys^a^	tK(UUU)mt	-^a^	TU2	10 849	10 777
tRNA-Leu_1^a^	tL(UAA)mt	+^a^	TU3	11 523	11 606
tRNA-Tyr^a^	tY(GUA)mt	+^a^	TU3	11 613	11 696
tRNA-His^a^	tH(GUG)mt	+^a^	TU3	11 702	11 774
tRNA-Thr^a^	tT(UGU)mt	+^a^	TU3	11 779	11 852
tRNA-Glu^a^	tE(UUC)mt	+^a^	TU3	11 855	11 926
tRNA-Ile	tI(GAU)mt	-	TU4	12 809	12 738
tRNA-Pro	tP(UGG)mt	-	TU4	14 017	13 944
tRNA-Cys	tC(GCA)mt	-	TU4	14 174	14 103
tRNA-Gly	tG(UCC)mt	-	TU4	14 251	14 180
tRNA-Arg_1	tR(UCU)mt	+	TU5	21 065	21 136
tRNA-Val	tV(UAC)mt	+	TU5	21 154	21 225
tRNA-Trp	tW(UCA)mt	+	TU5	21 227	21 298
tRNA-Phe	tF(GAA)mt	+	TU5	21 298	21 369
tRNA-Arg_2	tR(ACG)mt	+	TU5	21 407	21 478
tRNA-Met_1	tM(CAU)1mt	+	TU6	26 934	27 005
tRNA-Gln	tQ(UUG)mt	+	TU7	31 491	31 563
tRNA-Asp	tD(GUC)mt	+	TU7	31 569	31 641
tRNA-Ser_1	tS(GCU)mt	+	TU7	31 645	31 729
tRNA-Ser_2	tS(UGA)mt	+	TU8	31 924	32 005
tRNA-Leu_2	tL(UAG)mt	+	TU8	32 014	32 088
tRNA-Met_2^b^	tM(CAU)2mt ^b^	+	TU8	33 485	33 557

^a^These tRNAs are encoded by genes localized in the repeat sequence, another copy on the opposite strand is thus present in the reference sequence

^b^This tRNA displays high sequence similarity to tM(CAU)Q2 [32] of *S. cerevisiae*, so it is probably the initiator tRNA^fMet^, tM(CAU)1mt is therefore likely to be the regular tRNA^Met^

Interestingly, the gene encoding the RNA component of RNase P cannot be found in *C. albicans* mtDNA [11], suggesting that the enzyme is probably composed purely of protein subunits, like in many animal and plant organellar systems [102–104].

Transcription unit TU3 is the only one comprised exclusively of five tRNA genes, the remaining 19 tRNAs are contained within polycistronic units, comprising also rRNA and mRNA genes (Fig. 2b). Cleavage of precursors containing tRNA sequences by endonucleases RNase P and tRNase Z provides a likely mechanism liberating flanking transcripts, known as the tRNA punctuation model, first described in human mitochondria [12, 78]. In *C. albicans* mitochondria, tRNA punctuation is sufficient to account for all the cleavages necessary to produce the final mRNA and rRNA molecules. Excision of tRNAs liberates monocistronic transcripts of *RNL, COX2, COX3, ATP9, COX1, COB, RNS,* and *NAD4*, whereas *NAD6* with *NAD1*, *ATP8* with *ATP6*, *NAD2* with *NAD3*, and *NAD4L* with *NAD5,* would remain as co-translated bicistronic mRNAs (Figs. 1 and 2). The predicted open reading frames of *NAD* genes encoded on bicistronic transcripts are adjacent, and overlap by one nucleotide (the last A of the stop codon becomes A of the presumed ATG initiation codon), whereas *ATP8* and *ATP6* are separated by 125 nt of noncoding sequence. We used Northern blot hybridization to confirm the predicted sizes of these mature transcripts (Supplementary Results and Discussion, Additional file 1: Figure S4).

In *S. cerevisiae* an additional mechanism is responsible for processing at the 3’ termini of mRNAs by cleavage at a conserved AU-rich dodecamer sequence [18, 21, 32], whereas in *S. pombe* a C-rich stretch (the “C-core”) acts as the signal for the cleavage and maturation of the 3’ ends of mRNAs and the small subunit rRNA [19]. Additional putative cleavage signal sequences, consisting of GC rich 17-mers, were recently identified in *S. cerevisiae* [32]. In *C. albicans* we found no evidence for such mechanisms operating in addition to tRNA punctuation, which is sufficient to explain the generation of all observed mature transcripts and is therefore likely the sole mechanism responsible for fragmenting of primary transcripts into the final RNAs, like in the vertebrate system.

### Introns in the mitochondrial genes of *C. albicans*

**Fig. 5:**
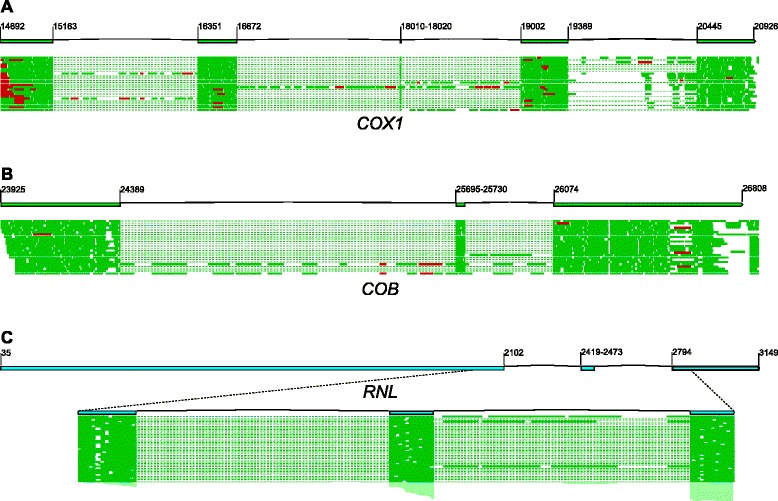
Split genes in the *C. albicans* mitochondrial genome. **a** Schematic exon-intron structure and the mapping of RNA sequencing reads in the *COX1* gene. **b** Schematic exon-intron structure and the mapping of RNA sequencing reads in the *COB* gene. **c** Schematic exon-intron structure and the mapping of RNA sequencing reads in the *RNL* gene. Exon coordinates are indicated (the first exon assumed to start with the +1 nucleotide of the transcript, as determined by RNA sequencing mapping and promoter identification), for very short exons the markers were joined for clarity of presentation. Dashed lines in the sequencing mapping represent reads that span the exon-exon junction, reads mapping to the forward (+) strand are shown in *green*, antisense (− strand) reads are shown in *red*. In (C) only the reads from the immediate vicinity of the two introns are shown as an inset for clarity. Green boxes represent protein-coding exons, *light blue* boxes are the rRNA exons. Gene structure and read mapping was visualized in CLC Genomics Suite 8 and edited for clarity

Introns are a common feature of organellar genes, and as mobile genetic elements they make an important contribution to the evolutionary dynamics of yeast mitochondrial genomes [29]. *In silico* analysis (http://www.candidagenome.org, [105]), based on the conservation of the protein-coding sequences, and similarity to orthologous sequences from *Candida parapsilosis* [35], indicated that in *C. albicans* there are four introns in the *COX1* gene, and two introns in the *COB* gene. Long-range mapping of RNA sequencing reads obtained in our experiments fully confirmed those predicted splicing sites, and provided first experimental evidence for the exon-intron structure of these two genes (Fig. 5). Additionally, we identified two introns in the large subunit rRNA (*RNL*) gene (Fig. 5c). These introns were not annotated in the reference genome, nor in the *Candida* Genome Database, but their presence was indicated in a complete genomic sequence of another *C. albicans* strain (CBS 562, [GenBank: KC993188.1], unpublished). This brings the total intron number in *C. albicans* mitochondrial genes to eight. The four introns of *COX1* are predicted to be group I introns by the *in silico* annotation server MFannot (http://megasun.bch.umontreal.ca/cgi-bin/mfannot/mfannotInterface.pl, [29]), whereas the two *COB* introns cannot be reliably assigned to a group by automatic prediction tools. MFannot predicts that the two introns in the *RNL* gene are also group I (IA) introns.

The four introns of *COX1* and the first of the two introns of *COB* contain ORFs in frame with the preceding exon. Such arrangement is often found in mitochondrial introns of Fungi, and it entails synthesis of a fusion protein encoded by the intron and the preceding exon(s) [32, 106, 107]. The intron-encoded proteins are involved in intron mobility (homing) and/or in splicing [108–114]. Analysis using the NCBI conserved domain database (CDD, [115]) revealed that all four putative proteins encoded by the introns of the *COX1* gene contain the LAGLIDADG endonuclease domain [116], that is found in proteins exhibiting RNA maturase (splicing), DNA endonuclease (homing), or both maturase and endonuclease activities. Sequence analysis alone cannot distinguish between these activities, as sometimes only a few amino acid substitutions determine whether a protein of this family acts as a maturase, endonuclease or a bifunctional maturase/endonuclease [117–119]. The protein encoded by the first intron of the *COB* gene contains signature motif of another class of homing endonucleases – the GYI-YIG superfamily [120].

### GC-rich elements in the *C. albicans* mitochondrial genome and transcriptome

The mitochondrial genomes of yeasts are generally AT-rich, and *C. albicans* (32 % GC) is no exception. The GC-rich fragments are often found dispersed in the genome as relatively short palindromic clusters that can potentially fold into hairpin structures [10, 26, 33–36, 121–124], and may also constitute mobile genetic elements and/or regulatory signals.

**Fig. 6:**
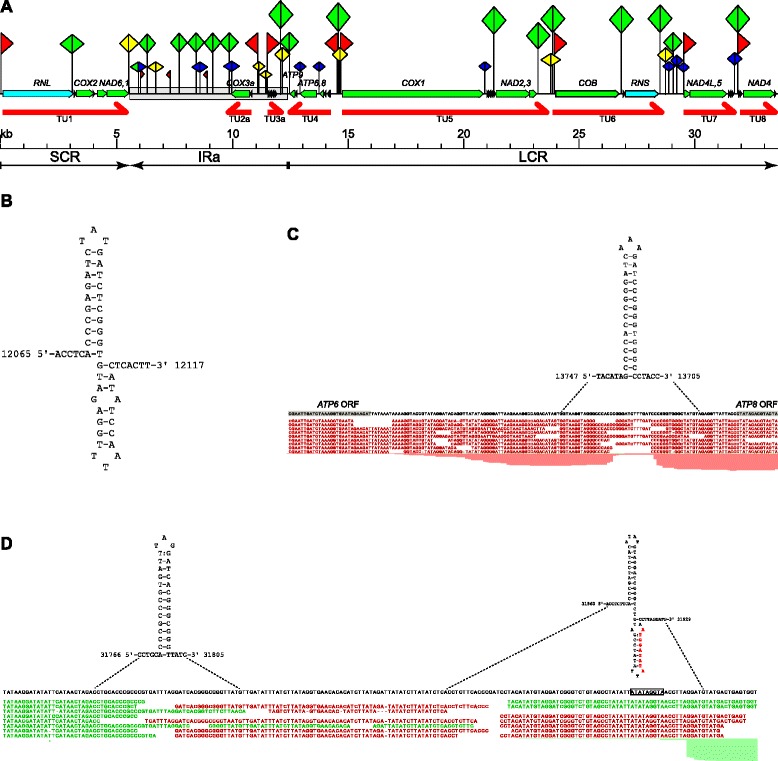
GC-rich elements in the *C. albicans* mitochondrial genome. **a** Schematic representation of the mitochondrial genome of *C. albicans* with known genes, major genomic regions and transcription units, annotated as in Figs. 1 and 2. The second inverted repeat region (IRb) is identical to IRa in sequence, and was omitted for clarity. Promoter sites are marked by red flags, hairpin elements are denoted as double triangles. *Green* double triangles are double hairpin (DHE-like) elements, *yellow* double triangles are DHE-like elements with an insertion between the two hairpins, and *blue* double triangles are single hairpins. **b** An example of the double-hairpin element located between transcription units TU3 and TU4. **c** A single hairpin structure in the noncoding region separating the *ATP8* and *ATP6* ORFs in a bicistronic mRNA. Sequencing reads mapped to the region are shown in *red*, *grey* rectangles highlight the end of *ATP8* ORF and the start of the *ATP6* ORF. This region is transcribed from the (−) strand of the reference genome, the sequence of the mRNA is thus a reverse complement of the shown reference. **d** GC-rich elements in the region between the end of transcription unit TU7 and the start of TU8. The promoter of TU8, forming part of the putative DHE-like element is shown in red in the structure, and is boxed in the reference sequence. Italics in the structure denote the nucleotides belonging to the tRNA-Ser_2 (tS(UGA)) sequence. Reads shown in *green* are sense strand reads, antisense reads are shown in *red* (both TU7 and TU8 are sense strand transcripts). Read mapping was visualized in CLC Genomics Suite 8 and edited for clarity

In *C. albicans* we identified about 40 such elements, dispersed throughout the entire mitochondrial genome, but generally localized outside coding sequences (Fig. 6, listed in Additional file 4: Table S4). The length and sequence of different GC rich sequence elements are variable, and their putative function (if any) can also be different. Tentatively, they can be grouped into three classes (Fig. 6a). Elements from the first group can be folded into a distinctive structure consisting of two adjacent stem-loops (Fig. 6b), resembling the double-hairpin elements (DHEs) identified in other fungal mitochondrial genomes, and considered to be mobile genetic elements [34, 122–125]. Another group resembles the DHEs, but the two stem loops are separated by a stretch of 40–60 nucleotides of AT-rich sequence that does not form a stable structure (an example is a structure in the unannotated putative transcript in the inverted repeat region, described in the next section). It is not clear whether they originated from the canonical DHEs by an insertion, or if they are unrelated. Finally, some GC-rich elements form a single stem-loop structure (Fig. 6c).

Some of the GC-rich hairpin forming sequences are located in the regions between the primary transcription units, whereas others are found within these units, but always between genes. Their presence could be related to the evolutionary history of the mitochondrial genome, where genes and groups of genes were often shuffled by recombination. Particularly, DHEs and DHE-like elements with insertions between the hairpins, could be related to the recombinational events shaping the evolution of mitochondrial genomes [34, 122–125].

A role in gene expression could be postulated for putative hairpin forming sequences located in or near the transcribed regions. Many such elements can be found in the intergenic regions separating the distinct primary transcription units, and thus they may play a role in transcription termination. For example, in the short region (190 bp) separating the last gene (tS(GCU)) of TU7 and the promoter of TU8, the forward mapping sequencing reads clearly stop at a GC-rich hairpin forming sequence (Fig. 6d), which thus appears to function as a terminator. Another GC-rich sequence, forming a double hairpin structure, is located in the region of TU8 promoter. GC-rich palindromes can also be found at the ends of other transcription units, as well as in the regions separating the opposite strand promoters of TU2 and TU3, as well as TU4 and TU5 (Fig. 6a). Such sequences can thus function as terminators and/or elements isolating the separate transcription units. Several GC-rich elements can also be found in the noncoding region of the inverted repeats, where we observed significant transcriptional activity (described in the next section).

Another potentially interesting example is the element found inside TU4, between the coding sequences of *ATP8* and *ATP6* (Fig. 6c). These two open reading frames are translated from a single bicistronic mature transcript, but unlike in the other bicistronic mRNAs, where the two genes are immediately adjacent to each other, there is a noncoding region of 125 nt separating the two ORFs. The GC-rich hairpin is located upstream of *ATP6,* and could be a target for a translational activator or a processing/stability factor.

### Transcripts of unknown function originating from inverted repeat regions

**Fig. 7:**
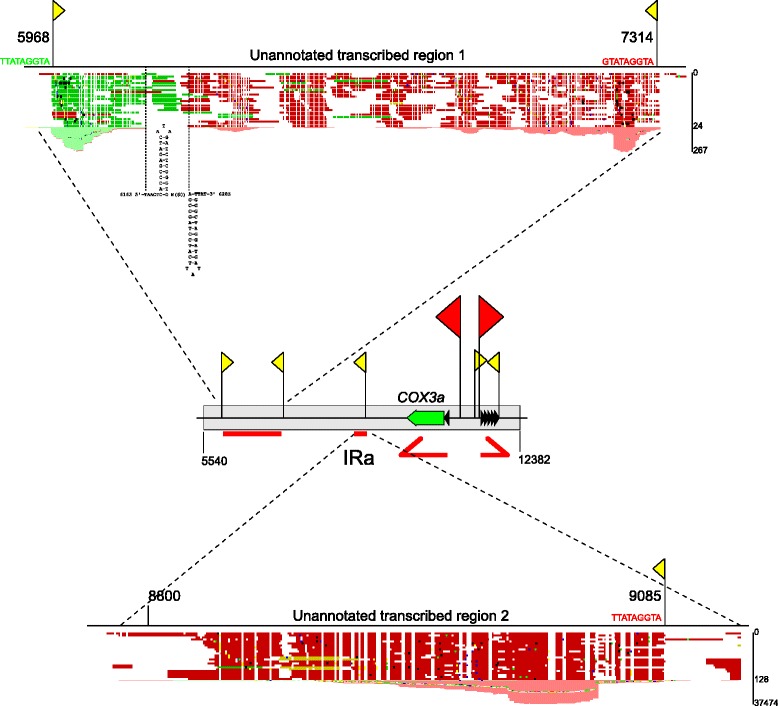
Putative transcripts in the inverted repeat region. The schematic representation of the inverted repeat (here IRa) region of the mitochondrial genome of *C. albicans*, annotated as in Figs. 1 and 2b, with unannotated transcriptionally active regions shown on insets. The positions correspond to the numbering in the reference sequence. Promoter sites are marked by flags: larger *red* flags denote main promoters for transcription units (confirmed by 5’-RACE), and *yellow* flags correspond to putative additional promoters with some support from RNA-seq reads (sequences conforming to the promoter consensus, but with no evidence for activity were omitted for clarity). The major primary transcription units (TUs) are shown as *red* arrows, and the putative transcripts of unknown function as red bars. Insets show sequencing reads mapping in the unannotated noncoding regions that show significant transcriptional activity. The promoter positions in the insets correspond to the +1 nucleotide. The consensus nonanucleotides of each promoter are shown as the sense strand sequence (reverse promoters are in *red*, while the forward strand promoter is in *green*). Bars on the right side of the insets provide the scale for number of sequencing reads mapped (note that these are not linear, and not to scale for the two regions). Read mapping was visualized in CLC Genomics Suite 8 and edited for clarity

In addition to transcripts mapping to the annotated genes of the *C. albicans* mitochondrial genome, we found significant transcriptional activity in the unannotated noncoding region of the inverted repeats. At least three putative promoter sequences could be responsible for driving transcription in this region (Figs. 7 and 2b). The first transcribed region, about 1300 bp long, is flanked by two promoter nonanucleotides (Fig. 7), one in the forward orientation, and the other in reverse. Both seem to be active, as the forward strand reads prevail immediately downstream of the first, forward promoter, while reverse orientation reads originate from the second, reverse strand promoter. Two GC-rich palindromic stem-loops, separated by 60 nt, can be found between the forward and reverse regions. The biological significance of this curious arrangement is currently unknown. Expression of this region is not particularly strong, but the number of reads that map there is significantly above the background. Transcription of the second region appears to be driven by a single reverse strand promoter, conforming to the nonanucleotide consensus (Fig. 7), and results in a short (~300 nt) putative RNA product. The core region of this transcript appears to be strongly expressed and stable, based on the very high number of RNA sequencing reads that map there.

Neither of these putative transcribed sequences contain any open reading frames, and they do not show significant homology to any of the known functional RNAs. It is therefore not clear, whether they represent *bona fide* functional or regulatory transcripts. The inverted repeats are associated with frequent recombination and replication [46, 61]. Even though replication initiation in *C. albicans* mitochondria is driven by homologous recombination, rather than by classical RNA priming, RNA:DNA duplexes are apparent in the mtDNA preparations, and can somehow be involved in the replication process [46].

### Evolutionary dynamics of transcriptional organization in yeast mitochondria

Mitochondrial genomes of yeasts, including the relatives of *C. albicans* grouped in the CTG clade, exhibit a great diversity in their genetic organization, in spite of a mostly conserved gene content [27, 28]. In order to investigate the effect of those rearrangements on the organization of gene expression units, we chose a set of mtDNA sequences of 11 CTG clade *Candida* species representative of different mitochondrial gene orders, 4 *Candida* species from early branching hemiascomycete lineages, with *Yarrowia lipolytica* as the early branch outgroup, inferred their phylogenetic relationships, and analyzed the alterations in gene order in the context of the primary transcription units inferred from our transcriptome study. The list of mtDNA sequences used in this comparison, including accession numbers and references is provided as supplementary data (Additional file 6: Table S6). The phylogenetic tree (Supplementary Results and Discussion, Additional file 1: Figure S5) is consistent with earlier hemiascomycete phylogenies [28, 126, 127] and shows a monophyletic CTG clade, with *C. vartiovaarae*, *C. norvegica, C. santjacobensis, C. salmanticensis*, and *Y. lipolytica* outside this clade as early branching lineages.

In general, this comparison indicates that the grouping of mitochondrial genes into polycistronic transcripts is not stable in evolution, and undergoes significant rearrangements even in relatively close species. There are, however, shorter blocks of synteny that seem to be conserved as they are shuffled into different combinations. One notable example is the pairing of *ATP8* and *ATP6,* translated from a single mRNA, which seems to be a common feature of all the mitochondrial genomes in hemiascomycetous yeasts. The synteny of these two genes extends beyond Fungi to vertebrate mtDNAs, but is not universal in Opisthokonts, as it is broken in the mitochondrial genomes of Schizosaccharomycetales [123] and Zygomycetes [125]. In mtDNAs of the analyzed yeast species we also observed that the genes encoding Complex I subunits: *NAD6* with *NAD1*, *NAD2* with *NAD3*, and *NAD4L* with *NAD5,* remain closely linked and probably co-translated from a single mature transcript, as the predicted ORFs either overlap (like in *C. albicans*) or are separated by only a few nucleotides. Unlike the *ATP8-ATP6* synteny, however, the *NAD* gene pairs are not conserved in mtDNAs of, for example, filamentous ascomycetes or Metazoa.

**Fig. 8:**
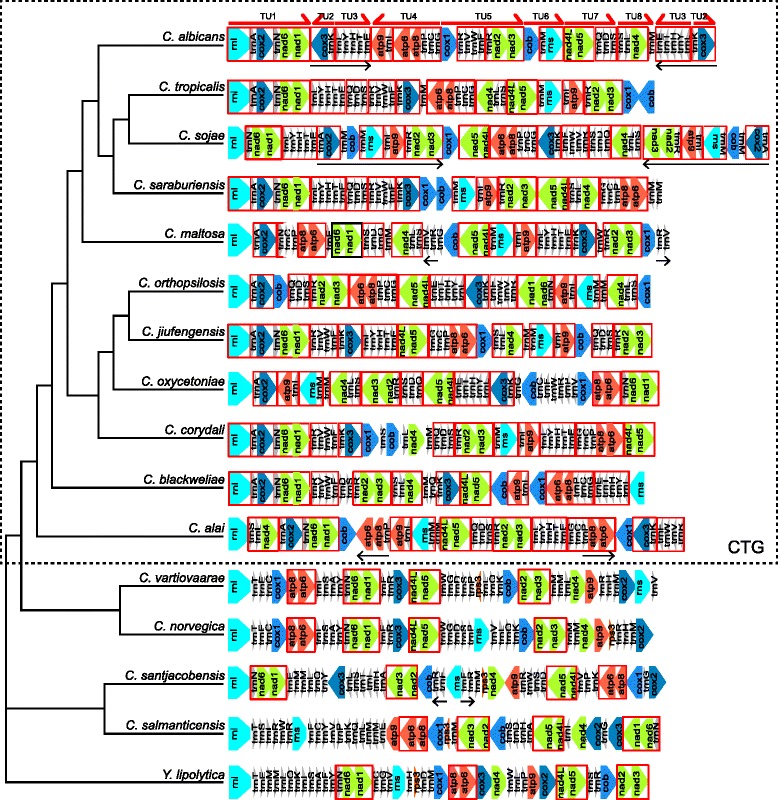
Evolution of the mitochondrial transcription units in *Candida*. A cladogram of 15 *Candida* species and *Yarrowia lipolytica* with the organization of mitochondrial genomes in each species represented schematically. The length of the symbols representing genes is arbitrary and not indicative of the actual length of respective sequence regions. Noncoding intergenic sequences are not shown. The cladogram is based on the tree inferred from mtDNA encoded protein (see Methods). The tree with meaningful branch lengths and posterior probabilities is available as a supplementary figure (Additional file 1: Figure S5). Species belonging to the CTG clade are boxed by a dashed line. For the purpose of gene order comparison, *RNL* was arbitrarily set as the first gene, regardless of the actual genome topology. Red boxes indicate sequence blocks that underwent rearrangements in the evolution of the analyzed genomes. Black arrows indicate repeat regions in the genomes, the major primary transcription units (TUs) identified in *C. albicans* are shown as red arrows

All the genes encoded in mtDNAs of *Y. lypolitica* and *C. norvegica* are transcribed from the same strand, and in *C. vartiovaareae* only the *rps3* gene (encoding a mitoribosome subunit, absent in the CTG clade yeasts mtDNAs) is reversed. With the exception of the *ATP8-ATP6* and *NAD* genes, the gene order in mtDNAs of the early branching yeasts shows no patterns of similarity to the members of the CTG clade. On the other hand, the mitochondrial genomes of CTG clade *Candida* species contain blocks of genes that are syntenic and correspond to blocks identified in previous studies [28, 35]. The way these blocks are assembled into transcription units seems, however, to highly variable (Fig. 8).

One such block corresponds to the first transcription unit of *C. albicans* mtDNA and consists of *RNL, trnA, COX2, trnN, NAD6,* and *NAD1*. It was identified as the ancestral gene order in the analysis of Valach et al. [28]. In some genomes it underwent further rearrangements, for example in *C. sojae* the *trnA, COX2* gene pair moved to a different location in the genome, whereas in *C. orthopsilosis, C. oxycetoniae* and *C. maltosa* the *trnN, NAD6, NAD1* block got transposed. Interestingly, in *C. alai* this block is interrupted by the insertion of another conserved block consisting of *trnS, trnL,* and *NAD4* which corresponds to *C. albicans* TU8. Only in *C. maltosa* the synteny of *trnN, NAD6, NAD1*, found even in the species not belonging to the CTG clade, and thus probably ancestral to all the analyzed mtDNAs, is broken.

Other examples of synteny include the blocks consisting of *trnG, trnC, trnP, ATP6,* and *ATP8* (*trnP, ATP6,* and *ATP8* were duplicated in *C. alai*); *trnK* and *COX3* (constituting the transcription unit TU2 of *C. albicans*); *trnI* and *ATP9*; *trnM* and *RNS* (not conserved in *C. alai*), and blocks consisting of tRNA genes, such as *trnL,Y,H,T,E* (TU3 of *C. albicans*), *trnQ,D,S,* and others (Fig. 8). The *COB* and *COX1* genes appear to undergo evolutionary shuffling on their own, independently of any other gene.

These evolutionary units of mitochondrial genome organization show only partial correlation with the primary transcription units. Whereas *C. albicans* TU1, TU2, TU3 and TU8 correspond to putative ancestral synteny blocks, TU4, TU5, TU6 and TU7 are composed of two or three of such blocks each (Fig. 8). The presence of secondary consensus promoters of uncertain activity inside the transcription units (Fig. 2b), as well as of GC-rich sequence elements (Fig. 6a) does coincide, however, with the boundaries of these blocks and is probably related to their evolution. The case of the DHE-like element upstream of tRNA-Met_1 sequence is particularly interesting. Downstream of the preceding *COB* gene, a DHE-like structure is followed by a nonanucleotide sequence (GTATAGGTA) closely related to the promoter consensus, and then the tRNA. RNA-seq reads indicate that these still belong to the same primary transcript, they could, however, show an evolutionary intermediate step that could lead to the formation of an independent transcription unit. Alternatively, it could be a vestige of a recent rearrangement that brought tRNA-Met_1 and *RNS* into this transcription unit. A similar arrangement can be observed in the region of TU8 promoter (Fig. 6d), where a DHE-like sequence includes the active promoter of TU8. This transcription unit is separated from the preceding transcript (TU7) by only a short intergenic stretch of about 140 bp that contains two GC-rich hairpin-forming elements. This suggests that a close link exists between evolutionary rearrangements mediated by the DHE-like elements and the reorganization of transcription in yeast mitochondrial genomes. Involvement of tRNA genes and their promoters is also a common theme in the evolutionary history of yeast mitochondrial genome organization.

## Conclusions

The mitochondrial genome organization shows great evolutionary variability, even in closely related organisms. As the promoter consensus sequences are short, and can be found at multiple sites in the genome often not corresponding to active transcription start sites, *in silico* analysis alone is not sufficient to draw conclusions about the number of active promoters. The study of the mitochondrial genome expression and its evolution must therefore involve transcriptome analysis in addition to mtDNA sequencing.

In the first experimental study of the expression of *Candida albicans* mitochondrial genome, we demonstrated that 14 protein coding genes, 24 tRNAs and 2 rRNAs are expressed as eight polycistronic primary transcription units that are processed into mature transcripts by excision of tRNAs. Introns are found in three of the genes (two in *LSU* rRNA, two in *COB* and four in *COX1*). Steady state levels of mature RNAs vary by orders of magnitude, even for sequences transcribed from the same promoter, suggesting RNA degradation as the main regulatory mechanism. The promoter consensus is a novel variant of the nonanucleotide sequence typical of yeast mitochondrial promoters. In addition to the main promoters driving the eight primary transcription units, there are multiple secondary promoter consensus sequences, that are either inactive, or could act as “boosters”. Their presence could be a vestige of former primary transcription start sites, that became redundant after new transcription units were formed. It may also facilitate the creation of new, separate transcription units after the extant polycistrons are broken up by genomic rearrangements. The fact, that the promoter sequences are short, and even significant departures from the consensus are tolerated, as evidenced by the active promoter of TU7 (Fig. 2), makes creation of new transcription start sites a likely evolutionary event.

It is clear that the polycistronic transcription units of yeast and animal mitochondria are not direct descendants of bacterial operons that can still be found in the mtDNA of jakobid protists [128, 129]. It has been suggested that the ancestral mitochondrial genome organization, at least in Opisthokonts, involved few promoters and very long primary transcripts [19]. This is the model of expression still found in Metazoa [96], *S. pombe* [19], and filamentous ascomycetes [92–95]. It could be also postulated for early branching Hemiascomycetes, like *Y. lipolytica*, as all the coding sequences are on the same genomic strand, there is, however, little experimental data about mitochondrial transcription in this organism [39].

Two primary transcripts were also suggested for *Candida parapsilosis* [35] and related species, this hypothesis is, however, based mostly on *in silico* analysis of the mtDNA sequence, and not on direct experimental study of transcription. Given the extraordinary evolutionary dynamics of the mitochondrial genome organization in yeasts, it is entirely possible that the putative two primary transcripts of *C. parapsilosis* are not directly descended from the ancestral state, but were formed as a result of rearrangements of multiple transcription units.

The main evolutionary force shaping the mitochondrial genomes of hemiascomycetous yeasts is the frequent recombination, constantly breaking apart and joining genes into primary transcription units. Even within the cells of the same species, as it was found in *C. albicans,* a mitochondrial genome can exist in forms differing in the gene order [46]*.* Long repeat regions [28] and GC-rich hairpin forming sequences [34, 122–125] are often involved in these recombinational events. Our study suggests that tRNA genes and their promoters, often with associated GC-rich elements, are also an important source of rearrangements. The conserved modules that undergo shuffling often start with a tRNA gene, as it is in the case of *trnA* and *COX2*; *trnK* and *COX3; trnI* and *ATP9*; *trnM* and *RNS; trnG, trnC, trnP, ATP6,* and *ATP8; trnS, trnL,* and *NAD4; trnN, NAD6,* and *NAD1,* and the groups consisting only of tRNA genes.

Short and promiscuous transcription initiation sites, apparent in yeast mtDNAs ensure that gene expression can adapt to the rapidly changing gene order. This means, however, that transcription initiation can have only a limited role in gene-specific regulatory mechanisms, and that posttranscriptional mechanisms should play a key role in the regulation of mitochondrial gene expression. The steady state levels of mature transcripts vary greatly among RNAs generated from the same primary transcript in *C. albicans* (Fig. 4), as well as in *S. cerevisiae* [32] and human [78] mitochondrial transcriptomes, and are most likely regulated by stability and degradation [32, 78, 97, 98], and possibly transcriptional attenuation [98]. Translational regulation (reviewed in [130]) is also an important mechanism controlling protein synthesis in yeast mitochondria. Entire families of proteins, such as the pentatricopeptide repeat (PPR) family, evolved in Eukaryotes as factors required to maintain and regulate the genetic system of the mitochondrial endosymbiont [131–135]. In order to gain a complete understanding of the evolution of the mitochondrial genetic systems, it will be necessary to follow the comparative genomics not only with the transcriptomic analysis, but also with an in-depth study of nucleo-mitochondrial interactions in a variety of taxonomically diverse organisms. *C. albicans* appears to be a good candidate for a new model system to study the functioning of the mitochondrial genome, and the overview of its transcriptome provides the necessary foundation for further investigations.

## Methods

### Strains and media

*Candida albicans* BWP17(*ura3*::*imm434*/*ura3*::*imm434; his1::hisG/his1::hisG; arg4::hisG/arg4::hisG*) [59] and SN148 (*arg4/arg4; leu2/leu2; his1/his; ura3::imm434/ura3::imm434; iro1:: imm434/iro1:: imm434*) [57] strains were grown in YPGal medium (1 % yeast extract, 2 % peptone and 2 % galactose) containing 80 μg/ml uridine at 37 °C until logarithmic growth phase.

### Mitochondria isolation and RNA preparation

Mitochondria were isolated from log-phase liquid culture by differential centrifugation as described previously [31]. Purified intact mitochondria were treated with 10 μg of RNase A (Thermo Scientific) at 37 °C for 5 min to remove co-purified cytoplasmic RNA. Isolation of mitochondrial RNA was performed from purified mitochondria using the hot phenol procedure [136] or by fenozol extraction (A&A Biotechnology).

Alternatively, total cellular RNA extracted using the hot phenol procedure [136] was treated with Ribo-Zero Gold rRNA Removal Kit (Yeast) (Epicentre) to deplete cytoplasmic rRNAs according to the manufacturer’s protocol, and used to construct total RNA libraries.

RNA samples were treated with DNAse (Roche) in the presence of RNase inhibitor (Ribolock, Thermo) in the manufacturer’s recommended buffer (Roche). DNA-free RNA was then extracted with phenol/chloroform/octanol (25:24:1), precipitated from the aqueous phase by ethanol/sodium acetate (pH 5.2) and dissolved in water. The quality of each preparation was assessed by conventional agarose gel electrophoresis and using BioAnalyzer.

### Library preparation and RNA-seq

RNA-seq libraries were prepared using the Ion Total RNA-Seq Kit v2 (Life Technologies) starting with 400–500 ng of either mitochondrial or total RNA, according to the manufacturer’s protocol. For mitochondrial RNA libraries the RNA fragmentation step was shortened to 4 min, preserving intact tRNAs. After the RNA fragmentation step each sample was divided into two equal aliquots, and one was treated with 0.5 U (mitochondrial RNA) or 0.75 U (total RNA) of tobacco acid pyrophospatase (TAP) (Epicentre Technologies) at 37 °C for 30 min. RNA quality and library construction was monitored using BioAnalyzer 2100 (Agilent Technologies) according to the manufacturer’s protocol.

The libraries were sequenced on the Ion Torrent Proton™ NGS System on a P1 chip using the Template OT2 200 Kit for template preparation and Ion PI™ Sequencing 200 Kit for sequencing (Life Technologies), all according to the manufacturer’s instructions. This method produces single-end, strand-specific reads. Raw sequencing data were processed using the Torrent Suite™ Software (Life Technologies). Barcode removal and quality trimming were performed in Torrent Suite™ using default parameters (30 % QC threshold, reads <25 nt rejected). The resulting reads range from 25 to 351–367 nt, with a mean of about 70 nt. The processed reads were exported as FASTQ files and imported into CLC Genomics Workbench 8 (http://www.clcbio.com) for the mapping, analysis and visualization of sequencing results. The RNA-seq workflow used in this software is based on the methodology of Mortazavi et al. [137]. The complete mtDNA sequence of *C. albicans* strain SC5314 [GenBank:AF285261.1], with additional feature annotations from the *Candida* Genome Database (Candida Genome Database, http://www.candidagenome.org, [105]) was used as the reference for read mapping. For analyses involving the nuclear genome, Assembly 22 of the *C. albicans* SC5314 genome sequence [138] was used as reference. Reads were mapped to both strands the entire reference sequence, including intergenic regions using default parameters (mismatch cost 2, indel cost 3, length and similarity fractions 0.8). Following the removal of the second copy of the inverted repeat region from the reference sequence, only uniquely mapping reads were counted. Expression values for annotated genes were calculated as RPKM [137].

### RT-PCR

Total cellular RNA was extracted and treated with DNAse as described above. 5 μg of DNA-free RNA was reverse transcribed by Maxima™ Reverse Transcriptase (Thermo Scientific) with random hexamers. 2 μl of tenfold diluted RT product was amplified in 28 PCR cycles in 20 μl with 0.5 U of Phusion polymerase (Thermo Scientific) and 10 pmol specific primers (Additional file 5: Table S5), and analyzed by agarose gel electrophoresis.

### Northern blot analysis

Northern hybridization was performed essentially as described previously [139]. 2.5 μg of mitochondrial RNA was separated on a 1 % denaturing formaldehyde gel, transferred onto Nytran N nylon membrane (GE Healthcare) and hybridized with the appropriate probe. Fragments of respective mitochondrial genes amplified by PCR and cloned into plasmid vectors were radiolabeled with α-^32^P-dATP using the Nick Translation System (Invitrogen). For the detection of *ATP6, ATP8, ATP9* oligonucleotide probes labeled with γ-^32^P-ATP using the T4 polynucleotide kinase (New England Biolabs) were used. All the probes used in Northern blot analysis are listed in Additional file 5: Table S5.

### 5’-RACE analysis of transcription start sites

Primary transcript 5’ termini were determined by 5’-RACE technique according to Bensing et al. [74] with minor modifications.

5’ triphosphates were converted to monophosphates by treatment of 5 μg of mitochondrial RNA with 10 units of tobacco acid pyrophospatase (TAP) (Epicentre Technologies) at 37 °C for 60 min in the presence of 40 U of RNase inhibitor (Thermo Scientific) in the appropriate buffer (50 mM sodium acetate pH 6.0, 10 mM EDTA, 1 % β-mercaptoethanol, 0.1 % TRITON X-100). Control RNA was incubated under the same conditions without TAP enzyme. RNA was then extracted with phenol/chloroform/octanol (25:24:1), precipitated from the aqueous phase by ethanol/sodium acetate (pH 5.2) and dissolved in water. RNA was subsequently ligated with 100 pM of chimeric DNA-RNA adapter (44 nt, blocked at the 5’ end with 5'-O-methyl-2'-deoxythymidine, last three nucleotides were RNA) at 17 °C for 12 h with 50 U of T4 RNA ligase (New England Biolabs), in the presence of 150 μM ATP and 80 U of RNase inhibitor (Thermo Scientific), in the appropriate buffer (50 mM Tris HCl, pH 7.5, 10 mM MgCl_2_, 4 mM DTT, 10 % DMSO). Ligation was followed by phenol/chloroform/octanol extraction, and ethanol/sodium acetate precipitation. RNA was dissolved in water and reverse-transcribed using 2 pmol gene specific primers and Super Script III Reverse Transcriptase (Invitrogen), according to the manufacturer’s instructions.

The products of reverse transcription were amplified by PCR, with 1 μl of RT reaction as a template, 25 pmol of each nested gene specific and adapter specific primer, 250 μM of each dNTP, 0.5 units of Phusion Polymerase (Thermo Scientific) in 25 μl of the manufacturer’s recommended buffer. Cycling conditions were 98 °C/3 min, 28 cycles of 98 °C/ 30 s, 55 °C/30 s, 72 °C/30 s, 72 °C/7 min. PCR products were separated on 3 % high resolution agarose gels (Polskie Agarozy), excised, eluted from the gel (Thermo Scientific GeneJET kit) and cloned into pJET1.2 Cloning Vector (Thermo Scientific CloneJET). Bacterial clones containing the plasmids with the inserts of proper size were identified by *Bgl*II digestion. Inserts were analyzed by Sanger sequencing with vector specific primers at the IBB PAS sequencing facility (http://oligo.ibb.waw.pl/).

Each 5’-RACE reaction was performed for two independent RNA preparations (from BWP17 and SN148 *Candida albicans* strains). A list of all oligos and primers used in 5’-RACE reactions is provided in Additional file 5: Table S5.

### Phylogenetic analysis

The mtDNA sequences used in the comparative analysis, with accession numbers and references are listed in Additional file 6: Table S6. Concatenated amino acid sequences encoded by 14 mitochondrial protein coding genes were aligned with MUSCLE version 3.8.31 [140] (3911 amino acid sites after manual removal of regions containing gaps in the alignment), and the tree was inferred using PhyloBayes (MPI version 1.5a) [141, 142] using the CAT-GTR model. Two MCMC chains were run in parallel for 5000 cycles, at which point the maximum discrepancy (maxdiff) value reached 0.009 (maxdiff <0.1 is considered sufficient). First 1000 trees in each chain were discarded as burnin, and one in two of the remaining trees were sampled for posterior consensus. The tree was rooted using the *Y. lipolytica* sequence as outgroup.

### Availability of supporting data

The datasets supporting the results of this article are available in the [Sequence Read Archive], [SRP056472 http://www.ncbi.nlm.nih.gov/Traces/study/?acc=SRP056472]

## Additional files

Additional file 1:
**Supplementary Results and Discussion (PDF).** Includes **Table S1.** and **Figures S1** **to S5**. (PDF 1524 kb) Additional file 2: Table S2. Promoters and promoter-like sequences in *C. albicans* mtDNA. Promoters of the primary transcription units are highlighted. In the “support” column “none” means that a consensus nonanucleotide is present, but no transcriptional activity can be reliably attributed to the promoter, “reads” means that there is an increased number of reads mapping downstream of the promoter, “reads and 5’-RACE” means promoters confirmed independently (Fig. 4). The second copy of inverted repeat (IRb) is omitted, as in Fig. 2b. (XLSX 9 kb) Additional file 3: Table S3. RPKM values of *C. albicans* mitochondrial transcripts. RNA preparations from purified mitochondria were used, reads were mapped to the reference genome with the second repeat (IRb) removed to ensure unambiguous mapping of transcripts originating in repeat regions. (XLSX 9 kb) Additional file 4: Table S4. GC-rich elements in *C. albicans* mtDNA. OpenDocument (ISO/IEC 26300) spreadsheet format. Colors denote different hairpin structure types, as in Fig. 8a. The second copy of inverted repeat (IRb) is omitted. (XLSX 9 kb) Additional file 5: Table S5. Primers, oligonucleotide probes and cloned PCR product probes used in this work. (XLSX 14 kb) Additional file 6: Table S6. Mitochondrial genome sequences used in the evolutionary comparisons. Genome size, accession numbers, number of introns, and references are provided. (XLSX 14 kb)

## Abbreviations

mtDNA: Mitochondrial DNA
OXPXOS: Oxidative phosphorylation
TU: Transcription unit
IR: Inverted repeat
DHE: Double-hairpin element
TAP: Tobacco alkaline phosphatase
RT-PCR: Reverse transcription polymerase chain reaction
5’ RACE: Rapid amplification of 5′ complementary DNA ends
NGS: Next generation sequencing
