# Supplementary material for: The transcriptome of Candida albicans mitochondria and the evolution of organellar transcription units in yeasts
Source: BMC Genomics. 2015 Oct 21;16:827. doi: 10.1186/s12864-015-2078-z (PMC4618339; doi:10.1186/s12864-015-2078-z)
Supplement: Additional file 1: — Supplementary Results and Discussion (PDF). Includes Table S1. and Figures S1 to S5. (PDF 1524 kb) [file 12864_2015_2078_MOESM1_ESM.pdf]

Supplementary results and discussion

RNA-seq analysis of *Candida albicans* mitochondrial transcriptome

Two commonly used laboratory strains of *C. albicans* (BWP17 and SN148) were cultured independently at 30°C for in rich medium and used to prepare RNA in all described sequencing experiments. RNA was prepared from mitochondria purified by differential centrifugation as described in Methods, treated with RNase-free DNAse, and the quality of each preparation was assessed by conventional agarose gel electrophoresis and using BioAnalyzer. In order to enrich the RNA sequencing products in reads corresponding to the 5' ends of primary transcripts, an aliquot of each RNA preparation was therefore treated with Tobacco Alkaline Phosphatase (TAP) prior to library preparation. Separate libraries were prepared from TAP-treated and untreated RNA, and the sequencing reads were analyzed separately. More reads mapping to regions immediately downstream of identified transcription start sites were observed in TAP-treated samples, but otherwise the results were very similar to those obtained without treatment with TAP.

The Ion Torrent Proton™ NGS System on a P1 chip using the Template OT2 200 Kit for template preparation and Ion PI™ Sequencing 200 Kit for sequencing (Life Technologies) were used in RNA-seq. This method produces single-end, strand-specific reads. After trimming of the barcode sequences and rejecting reads shorter than 25 nt, the resulting reads ranged from 25 nt to 351-368 nt. The mean and median read lengths were around 70 nt, reflecting the abundance of tRNA sequences in each library. With the exception of the tRNA peak around 70 nt, the lengths of the remaining reads exhibited a distribution close to normal.

In principle, multiple incubation and centrifugation steps in the mitochondrial purification protocol could influence the stability of short-lived RNA species, like the unprocessed precursors. In order to account for this, we prepared additional libraries from total *C. albicans* RNA isolated using a rapid protocol (see Methods for details). Obviously, using total RNA for library construction and mapping the resulting reads to the mitochondrial genome, results in a much lower mapped to total reads ratio, but still provides enough mapped reads for in-depth transcriptome analysis. Whereas some quantitative differences in read coverage could be noticed, the general picture observed using a total RNA preparation and RNA obtained from purified mitochondria was similar. The main difference is that the strongest tRNA peaks are significantly lower in RNA-seq from total RNA preparation, and thus other RNAs appear more prominent in comparison. On the other hand, more reads mapping to rRNAs are apparent in sequencing results from total RNA preparations. These differences may partially be due to a slightly more aggressive digestion step in the protocol for total RNA sequencing (see Methods) that decreases the signal from short abundant RNAs, like tRNAs. Reads mapping to the noncoding unannotat-

ed regions in the inverted repeats also exhibit quantitative differences in RNA-seq coverage in libraries from total and mitochondrial RNA preparations.

The basic statistics of the RNA sequencing and mapping of the *C. albicans* mitochondrial transcriptome are summarised in Table 1.

Table 1. Statistics of RNA sequencing of the *C. albicans* mitochondrial transcriptome

Experiment[a]	Total reads[b]	Mapped reads	% reads mapped to mtDNA
Mitochondrial RNA, untreated	16 002 239	9 259 151	57.9%
Mitochondrial RNA, TAP treated	9 253 487	6 960 015	75.2%
Total RNA, untreated	30 269 485	4 172 022	13.78%
Total RNA, TAP treated	37 527 436	3 891 812	10.4%

[a]Each experiment consisted of two libraries from separately cultured strains (BWP17 and SN148), reads were pooled for analysis, as no differences between strains were observed.

[b]After trimming of adapter sequences.

Overall, however, the repeatability of the quantitative RNA-seq analysis between different preparations is very good, particularly for annotated features. When reads from two *C. albicans* strains (BWP17 and SN148) were counted separately as two independent experiments, expressed as RPKM and compared, the reproducibility of the obtained expression data was very high (r = 0.97) (Figure S1). Similarly, treatment with Tobacco Alkaline Phosphatase (TAP) did not affect the estimated transcript levels (r = 0.98) (Figure S1). Results obtained using total cellular RNA preparations compared to RNA isolated from pure mitochondria show more discrepancies (r = 0.68), that are mainly due to tRNAs being underrepresented in experiments using total RNA; data for protein coding genes agree much better (r = 0.84) despite significant differences in the two RNA isolation protocols (Figure S1). Overall these results show that the quantitative expression data reported here are highly reproducible.

The majority (>90%) of reads from the mitochondrial RNA preparations that did not map to the mtDNA reference could be unambiguously mapped to the nuclear genome sequence, mostly to the highly expressed rRNA and tRNA genes. When reads from the mitochondrial

preparation mapping to the nuclear genome were compared to the reads from total RNA preparations, we noticed only a moderate enrichment in cytoplasmic rRNA sequences (25S, 18S and 5S rRNA). This is consistent with copurification of cytoplasmic ribosomes with mitochondria that was also observed in the human mitochondrial transcriptome study (Mercer et al. 2011). It is still possible that some nuclear-encoded transcripts are imported into *C. albicans* mitochondria, but identifying them would require a different experimental setup, involving removal of the outer membrane and IMS and analysis of enrichment of RNA-seq reads mapping to the nuclear genome in the resulting mitoplast preparation in comparison to the libraries obtained from intact mitochondria. Such analysis is beyond the scope of the present study, which is focused chiefly on the expression of the mitochondrial genome.

Verification of primary transcription unit continuity by RT-PCR

As the RNA-seq coverage drops significantly in some regions of the putative transcription units, while a few reads map to intergenic sequences outside the identified transcripts, it is important to obtain independent verification of the proposed transcription units and start sites. We used RT-PCR to verify the continuity of identified transcription units.

In RT-PCR we obtained amplification products with primers annealing to *RNL* and *NAD6*, thus confirming the continuity of the TU1 primary transcript, *COX1* and tRNA-Val, as well as *COX1* and *NAD2,* confirming the continuity of TU5, and tRNA-Ser_2 and tRNA-Met_2, confirming the continuity of TU8. We failed to obtain detectable amplification products with primers annealing to *RNS* and *NAD4L*, indicating that TU7 is a separate transcription unit from TU6, *NAD3* and *COB*, indicating that TU5 is a separate transcription unit from TU6. All primer sequences are listed in Additional File 5 (Table S5).

No evidence of transcription initiation at the additional promoters inside the primary transcription units

Additional sequences conforming to the promoter nonanucleotide consensus were also found inside the established primary transcription units (Figure 2B). Some of them are in the sense orientation, are located upstream of highly expressed tRNA and rRNA sequences, and could thus contribute to variations in the steady state level of different transcripts originating from the same primary transcription unit. Using 5'-RACE, we attempted to confirm the activity of two such putative promoters, upstream of tRNA-Arg_1 (tR(UCU)) and *RNS*. Whereas a product of the expected size was amplified in both cases, no clear differences between TAP-treated and untreated samples were apparent (Figure S3); the 5' ends of these molecules can thus

be attributed to the processing of a primary transcript rather than independent transcription initiation.

Verification of the mature transcripts by Northern blots

We used Northern blots in order to confirm the existence of mature mRNA and rRNA species predicted by the RNA-seq analysis. RNA was prepared from purified mitochondria of *C. albicans* (strain BWP17) and separated by agarose/formaldehyde gel electrophoresis in denaturing conditions. Bicistronic, and monocistronic RNAs were visualized by hybridization to gene-specific probes.

The results confirmed the existence of the four bicistronic mRNAs as major RNA species, with only a very small amount of *NAD5, NAD2* and *ATP8* signal corresponding to a single, monocistronic form (Figure S4A). The probes corresponding to the remaining six mRNAs (*COX2, COX3, ATP9, COX1, COB* and *NAD4*) and two rRNAs (*RNL* and *RNS*) hybridized to bands corresponding in size to the predicted monocistronic transcripts (Figure S4B).

Northern blot also confirmed the presence of the two introns in the large subunit rRNA (*RNL*) gene, as the estimated size of the mature transcript observed on Northern blots (~2500 nt, Figure S4B), which corresponds to the predicted size of spliced exons (2466 nt), but not to the entire gene (>3100 nt). This was further confirmed by RT-PCR with primers localised in the 3' region of the first exon, and the 5' region of the third exon, wherein a single product corresponding to the fully processed transcript was obtained, indicating that the splicing reactions are rapid, and/or that unspliced precursors are efficiently degraded. Only in the case of *COB* precursors corresponding to the splicing intermediates were clearly visible on the Northern blot (Fig S4B).

Supplementary figures

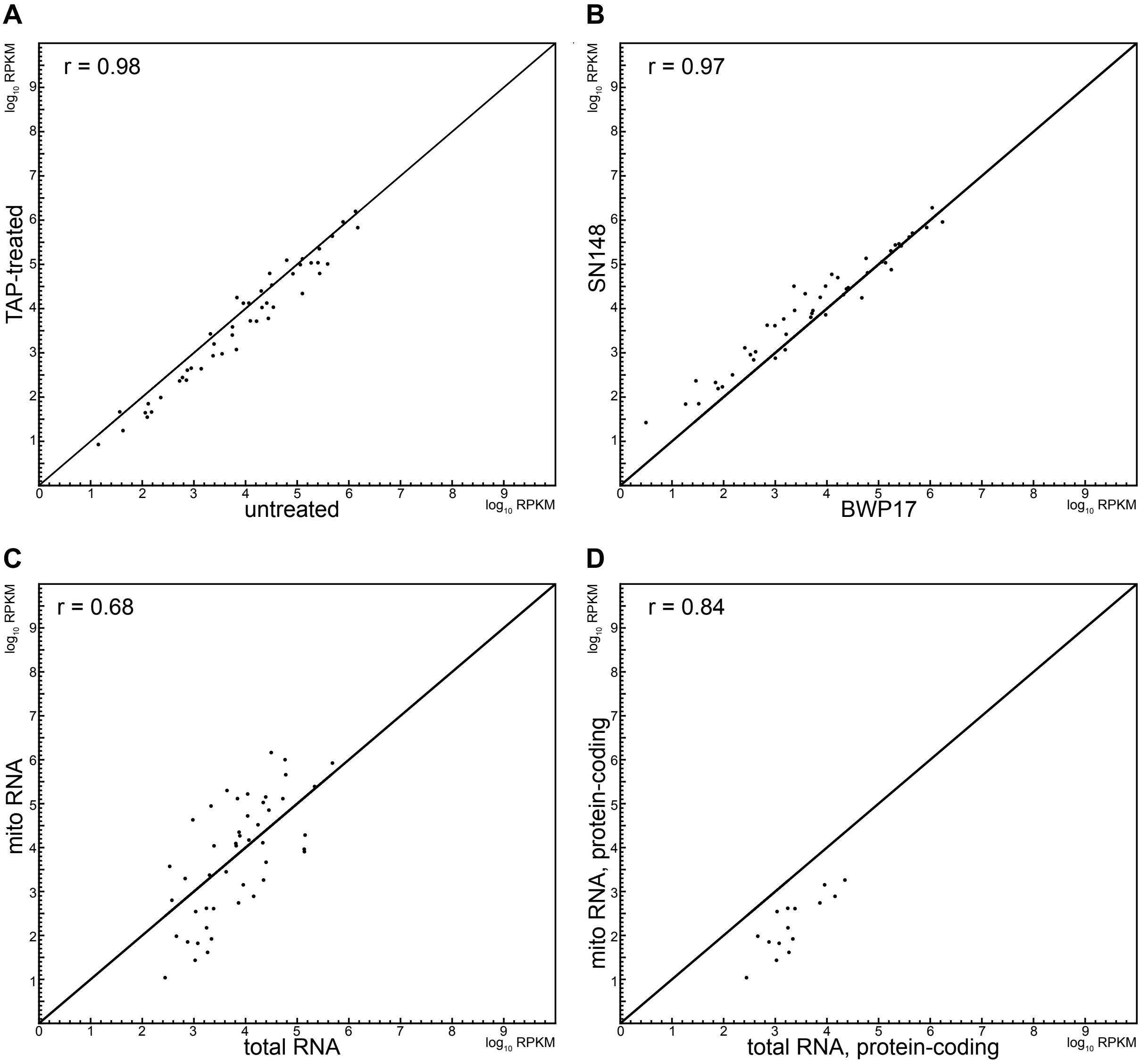

Figure S1. Scatterplots showing the correlation of mitochondrial gene expression values in different RNA sequencing experiments. Log_{10} transformed RPKM values are shown. (A) Comparison of expression values (in RPKM) between mitochondrial RNA preparations treated with Tobacco Alkaline Phosphatase (TAP) and untreated. (B) Comparison of expression values (in RPKM) between mitochondrial RNA preparations from BWP17 and SN148 strains. The two strains were grown as separate cultures, and the mitochondrial RNA isolation, library preparation, and sequencing were performed independently. (C) Comparison of expression values (in RPKM) between mitochondrial RNA preparations and total RNA preparations. (D) Comparison of expression values (in RPKM) between mitochondrial RNA preparations and total RNA preparations, limited to protein-coding transcripts. Comparison of expression values for annotated genes was performed and visualized as scatter plots in CLC Genomics Suite 8.

Supplementary figures

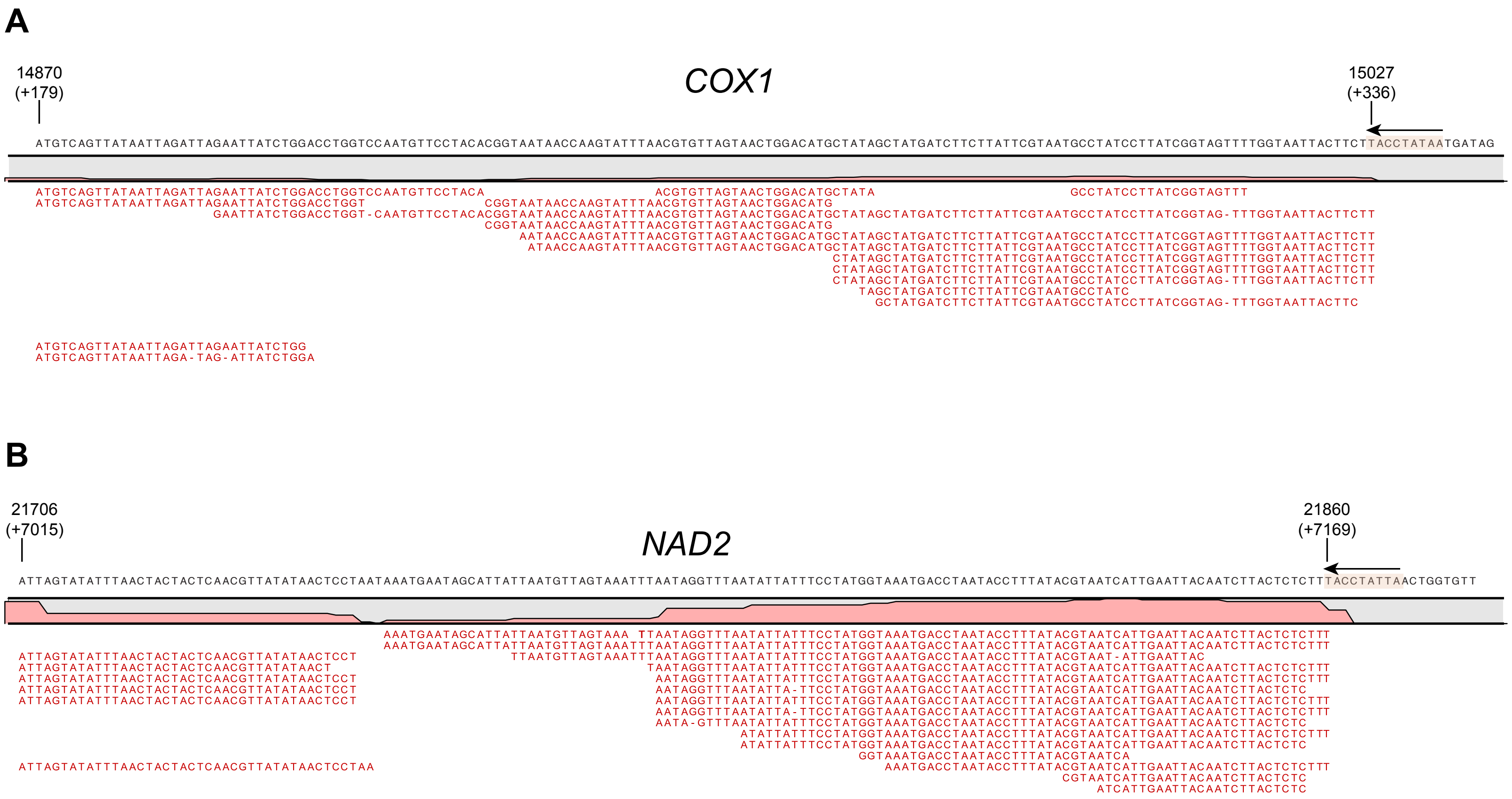

Figure S2. Examples of antisense transcripts generated by reverse orientation promoters in the COX1 (A) and NAD2 (B) genes. Genomic reference sequence (forward strand) is shown with antisense reads (red) mapped to the reverse strand of the reference. Forward (sense) reads are not visible in this analysis. Numbers denote positions in the reference sequence, numbers in parentheses are relative to the transcription start of the primary TU5 transcript. The reverse promoters are marked with a box and an arrow. The promoter sequences correspond to the consensus TTATAGGTA (A) or TAATAGGTA (B) on the complementary strand. Similar antisense promoters with associated sequencing reads can also be found in other locations in the genome (Figure 2). Gene structure and read mapping was visualized in CLC Genomics Suite 8 and edited for clarity.

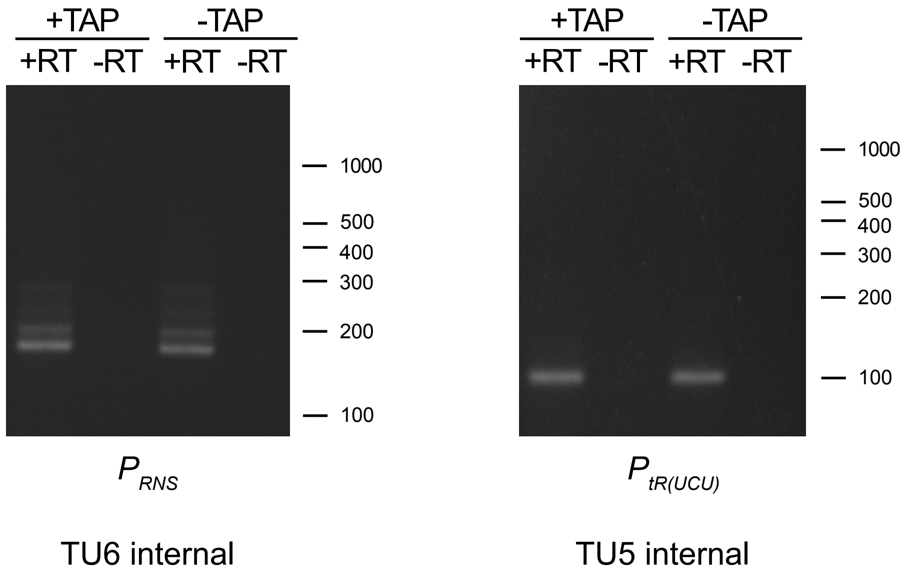

Figure S3. 5'-RACE analysis of putative internal promoters of TU5 and TU6. 5'-RACE products amplified from TAP-treated and untreated RNA preparations from purified *C. albicans* mitochondria were separated on 3% high resolution agarose gels alongside molecular weight markers (O' Gene Ruler DNA Ladder Mix, Thermo) and visualized by ethidium bromide staining. Positions of bands from the molecular weight markers are indicated on the left side of each gel.

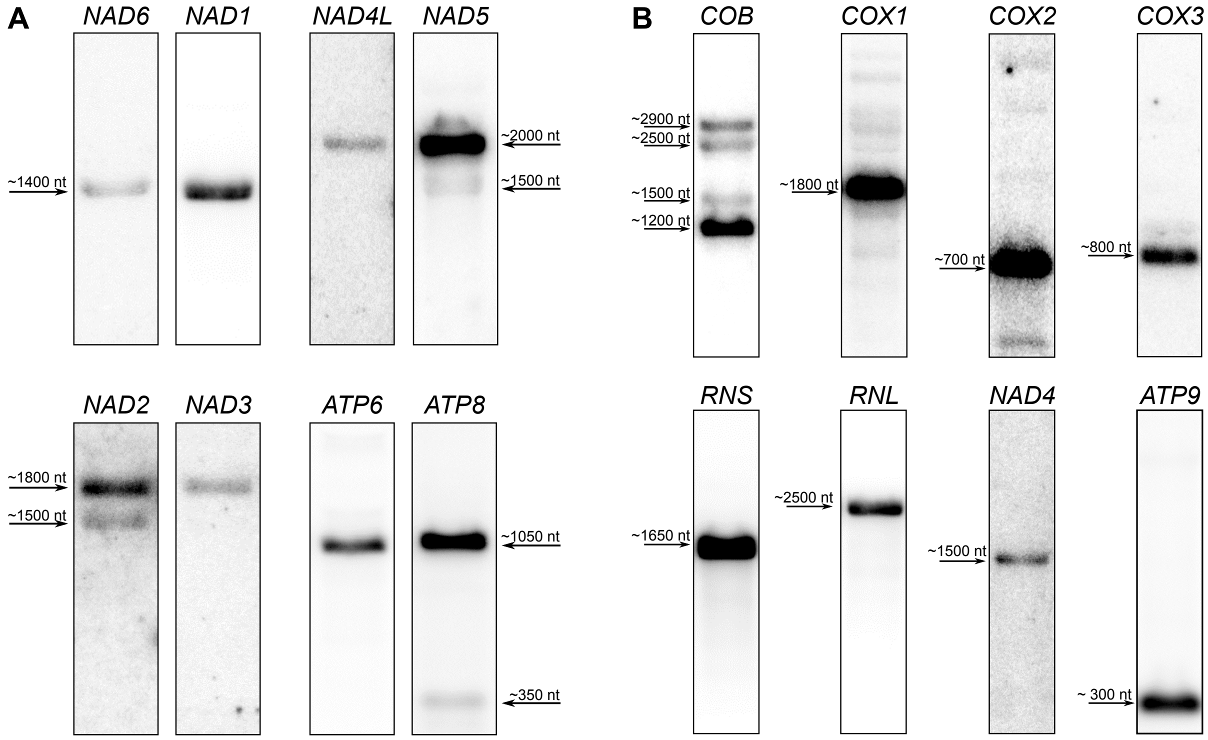

~

Figure S4. Northern blot analysis of mRNA and rRNA mature transcripts of C. albicans mitochondria. (A) Bicistronic, and (B) monocistronic RNAs were visualized by Northern blot and hybridization of RNA prepared from purified mitochondria of *C. albicans* (strain BWP17) and separated by agarose/formaldehyde gel electrophoresis in denaturing conditions. The probes were radiolabeled cloned PCR products, or in the case of *ATP6, ATP8,* and *ATP9,* synthetic oligonucleotides. Sizes of hybridizing bands were estimated by comparison to the migration of RiboRuler High Range RNA Ladder (Thermo Scientific) visualized by staining with methylene blue (not shown). The probes are described in Additional file 5: Table S5.

Supplementary figures

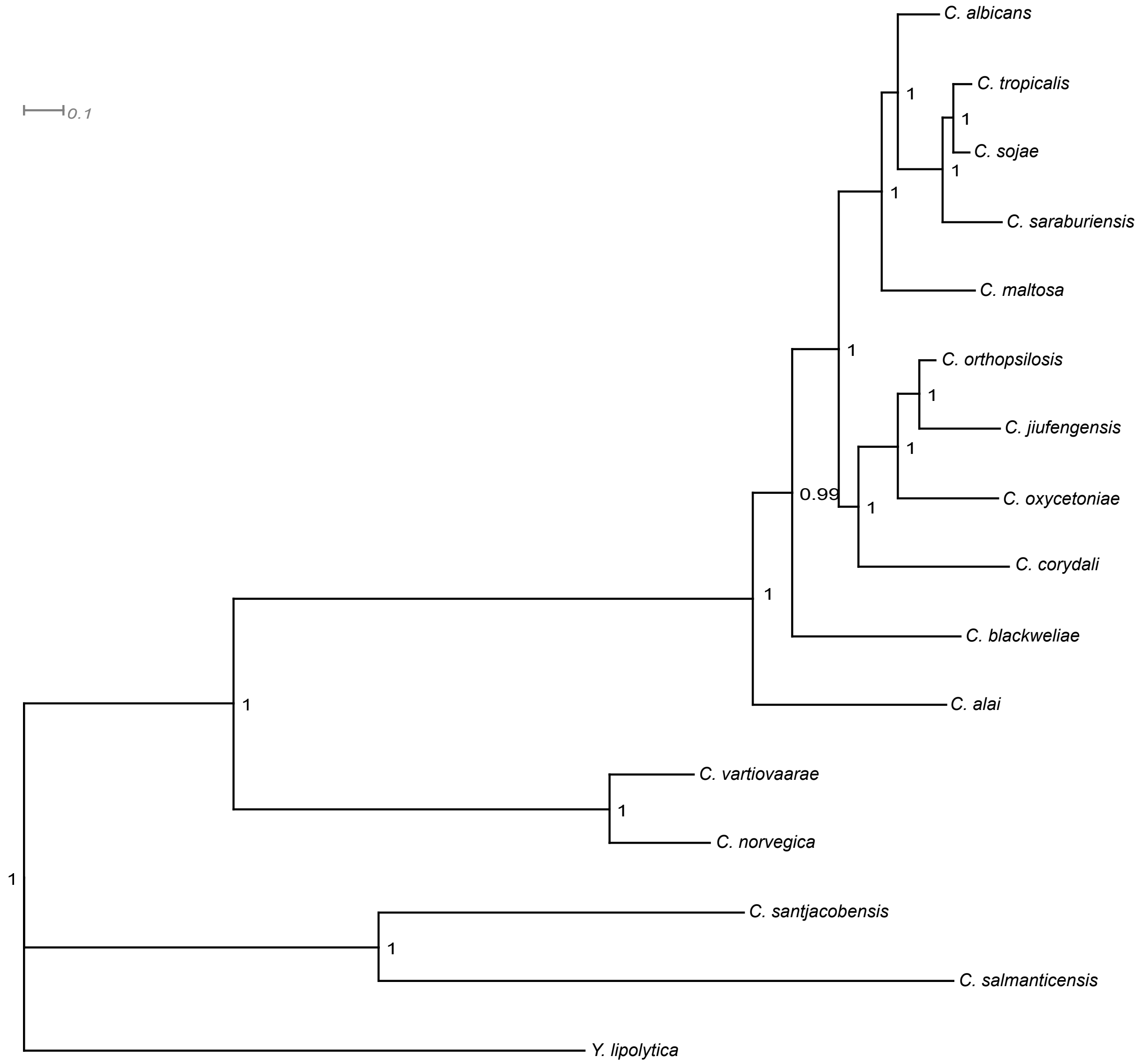

Figure S5. Phylogenetic tree of 15 *Candida* species and *Y. lipolytica* based on mtDNA encoded proteins. The mtDNA sequences are listed in Additional file 6: Table S6. The protein sequences were aligned with MUSCLE and the tree was inferred using PhyloBayes as described in Methods. The values at nodes are the posterior probabilities. This tree was used to make the cladogram in Fig 8.
